# Don't throw out the sympatric speciation with the crater lake water: fine‐scale investigation of introgression provides equivocal support for causal role of secondary gene flow in one of the clearest examples of sympatric speciation

**DOI:** 10.1002/evl3.78

**Published:** 2018-08-15

**Authors:** Emilie J. Richards, Jelmer W. Poelstra, Christopher H. Martin

**Affiliations:** ^1^ Biology Department University of North Carolina at Chapel Hill Chapel Hill North Carolina 27599; ^2^ Biology Department Duke University Durham North Carolina 27710

**Keywords:** adaptive radiation, gene flow, introgression, population genetics, speciation genomics, sympatric speciation

## Abstract

Genomic data has revealed complex histories of colonization and repeated gene flow previously unrecognized in some of the most celebrated examples of sympatric speciation and radiation. However, much of the evidence for secondary gene flow into these radiations comes from summary statistics calculated from sparse genomic sampling without knowledge of which specific genomic regions introgressed. This tells us little about how gene flow potentially influenced sympatric diversification. Here, we investigated whole genomes of Barombi Mbo crater lake cichlids for fine‐scale patterns of introgression with neighboring riverine cichlid populations. We found evidence of secondary gene flow into the radiation scattered across <0.24% of the genome; however, from our analyses, it is not clear if the functional diversity in these regions contributed to the ecological, sexual, and morphological diversity found in the lake. Unlike similar studies, we found no obvious candidate genes for adaptive introgression and we cannot rule out that secondary gene flow was predominantly neutral with respect to the diversification process. We also found evidence for differential assortment of ancestral polymorphisms found in riverine populations between sympatric sister species, suggesting the presence of an ancestral hybrid swarm. Although the history of gene flow and colonization is more complicated than previously assumed, the lack of compelling evidence for secondary gene flow's role in species diversification suggests that we should not yet rule out one of the most celebrated examples of sympatric speciation in nature without a more thorough investigation of the timing and functional role of each introgressed region.

**Impact Statement**

Sympatric speciation, the evolution of reproductive isolation without the aid of geographic barriers, is a fascinating process for its illustration of the power of natural and sexual selection alone to create new species. Despite exhaustive searches, only a handful of case studies provide convincing evidence for sympatric speciation in nature. Even in some of the clearest examples of sympatric speciation, the use of genomic data has revealed more complicated histories of gene flow from geographically separated populations than once thought. Evidence for these complicated histories is typically collected as a single point estimate of the entire genome, which can tell us that there was secondary gene flow but nothing about how that gene flow may have contributed to the evolution of new species. Here, we exhaustively search whole genomes for signatures of secondary gene flow into the adaptive radiation of Barombi Mbo crater lake cichlids, one of the clearest case studies of sympatric speciation that has recently come under doubt. We characterized genetic and functional diversity in regions of the genome that have experienced gene flow differently among the species to determine the role of gene flow in the speciation process. Very few regions of the genome appear to have experienced differential gene flow and there was no clear evidence that gene flow after initial diversification in the lake brought in essential adaptive genetic variation for ecological and morphological diversity. We conclude that multiple colonizations of the lake before diversification began (i.e., a hybrid swarm) may have contributed more to the radiation than secondary gene flow after initial diversification. We still cannot rule out the possibility of sympatric speciation in one of the most celebrated examples in nature.

## Introduction

Sympatric speciation, the endpoint on the speciation‐with‐gene‐flow continuum, is defined as the evolution of reproductive isolation without the aid of geographic barriers under complete panmixia and constant gene flow between diverging populations (Coyne and Orr 2004; Fitzpatrick et al. 2008; Mallet et al. 2009). Sympatric speciation has fascinated evolutionary biologists since Darwin for its illustration of the power of complex interactions between natural and sexual selection to create new species. Despite intense searches, very few case studies have been able to meet the rigorous criteria for demonstrating sympatric speciation in nature (Coyne and Orr 2004; Bolnick and Fitzpatrick 2007). Even in some of the more convincing examples that do meet these criteria, genomic data have revealed more complex evolutionary histories of multiple colonization and repeated gene flow than previously assumed (Papadopolus et al. 2011; The Heliconius Genome Consortium et al. 2012; Geiger et al. 2013; Alcaide et al. 2014; Igea et al. 2015; Malinsky et al. 2015; Martin et al. 2015a; Kautt et al. 2016; Poelstra et al. 2018).

However, much of the support for complicated histories involving repeated gene flow into sympatric radiations comes from genome‐wide tests for gene flow (e.g., Lamichhaney et al. 2015; Martin et al. 2015a; Meier et al. 2017). One prediction of models of speciation with gene flow is that divergence between incipient species should be heterogeneous across the genome (Nosil et al. 2008; Feder et al. 2012; Nosil and Feder 2012). Indeed, high heterogeneity in genomic differentiation has been found across the genomes of many recent or incipient sister species (e.g., Jones et al. 2012; Martin et al. 2013; Poelstra et al. 2014; Soria‐Carrasco et al. 2014; Malinsky et al. 2015; McGirr and Martin 2016), although other processes besides differential gene flow across the genome can produce similar heterogeneous patterns (Noor and Bennett 2009; Nachman and Payseur 2012; Cutter and Payseur 2013; Cruickshank and Hahn 2014; Guerrero and Hahn 2017; Ravinet et al. 2017). Only a handful of genes may directly contribute to the speciation process whereas the rest of the genome is porous to gene flow while reproductive isolation is incomplete (Wu 2001; Wu and Ting 2004). Therefore, gene flow detected at the genome‐wide level from populations outside the sympatric radiation does not by itself constitute evidence that secondary gene flow was involved in the divergence process among incipient species and shaped the radiation.

The Cameroon crater lake cichlid radiations are some of the most compelling cases for sympatric speciation in the wild (Coyne and Orr 2004; Bolnick and Fitzpatrick 2007; Martin 2012). The most speciose of these radiations is found in the isolated 2.3 km wide volcanic crater lake Barombi Mbo (Trewavas et al. 1972; Schliewen et al. 1994; Schliewen and Klee 2004). Barombi Mbo hosts a radiation of 11 endemic cichlid species, many of which have clear morphological and ecological separation from other sympatric species (Schliewen et al. 1994). Some endemics have evolved unique specializations, such as the spongivore *Pungu maclareni*, seasonal lekking species *Myaka myaka* (C.H.M. pers. obs.), and the deep‐water hypoxia specialist *Konia dikume* (Trewavas et al. 1972). Other endemic species, such as *Stomatepia mariae* and *S. pindu*, appear to be incipient or stalled species complexes with only slight morphological and ecological divergence at the extremes of a unimodal distribution of phenotypes (Martin 2012). However, evidence of differential introgression, weak support for Barombi Mbo monophyly, and differences in levels of shared ancestry with outgroup riverine populations from genome‐wide RADseq data all suggest secondary gene flow into the radiation after the initial colonization, casting doubt on one of the best examples of sympatric speciation in the wild (Martin et al. 2015a).

Here, we dissect those signals of repeated gene flow to investigate their role in the radiation using whole‐genome sequences. We performed exhaustive searches for all genetic patterns consistent with secondary gene flow into the ancestral Barombi Mbo population or into subclades after their initial divergence. We used a machine learning approach to finely dissect and partition different phylogenetic signals across the genome and sliding window genomic scans to test for differential introgression. We found evidence of both shared introgression between sister species and across subclades in the radiation as well as differential introgression among sister species across small regions of the genome. However, functional and genetic diversity in these regions do not paint a clear picture of how introgressed variants may have contributed to speciation in these groups. Our results suggest that either 1) rare introgression of variants in poorly characterized genetic pathways contributed to the morphological and ecological diversity of the radiation (speciation with an allopatric phase), 2) secondary gene flow was predominantly or completely neutral and did not contribute to diversification in Barombi Mbo (sympatric speciation with gene flow), or 3) multiple colonizations of the lake before diversification brought in genetic variation that was then differentially sorted among incipient species (sympatric speciation from a hybrid swarm).

## Methods

### SAMPLING AND GENOME SEQUENCING

**Figure 1:**
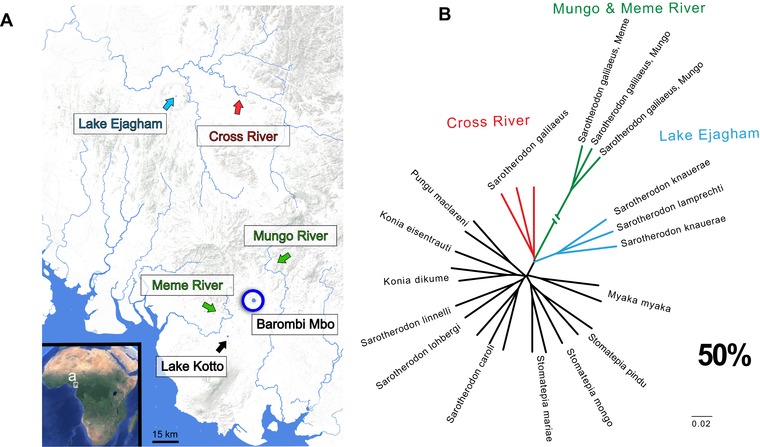
**The predominant phylogenetic relationship among Barombi Mbo radiation and neighboring riverine populations**. A) Map of lakes and riverine drainages within the volcanic belt of Cameroon in the Northwest and Southwest provinces (Ambazonia). Modified from Figure 2 in Martin et al. (2015a). B) The topology assigned to the largest percentage of the genomes across Barombi Mbo radiation and outgroup *Sarotherodon* species. Across most of the genome Barombi Mbo species (black) are more closely related to each other than riverine outgroup populations of *S. galilaeus* Mungo and Meme River (green) and *S. galilaeus* Cross River (red), or the Lake Ejagham *Sarotherodon* radiation (blue).

We sequenced whole genomes of one to three individuals from 10 out of the 11 species within the sympatric radiation of Oreochromine cichlids in Cameroon crater lake Barombi Mbo (excluding *Sarotherodon steinbachi*, which is morphologically and ecologically similar to the other three *Sarotherodon* species), an endemic *Sarotherodon* species pair from Lake Ejagham, and outgroup *Sarotherodon* individuals from all three river drainages surrounding the lake: Meme, Mungo, and Cross rivers (Fig. 1A). Details on the collection, extraction, alignment to the *Oreochromis niloticus* reference genome (Brawand et al. 2014), and variant calling protocols following the standard GATK pipeline (McKenna et al. 2010) are provided in the supplementary methods.

### CHARACTERIZATION OF INTROGRESSION PATTERNS ACROSS THE GENOME

We exhaustively searched our genomic dataset for patterns of non‐monophyletic Barombi Mbo relationships using the machine learning program *Saguaro* (Zamani et al. 2013) to identify regions of the genome that supported phylogenetic topologies consistent with expectations from multiple colonizations and secondary gene flow into the radiation (i.e., paraphyletic/polyphyletic Barombi Mbo radiations). This method infers relationships among individuals in the form of genetic distance matrices and assigns segments across the genomes to different topologies without a *priori* hypotheses about these relationships. We partitioned the genome into a total of 75 unique topologies (well past the inflection point at 30 topologies in which the percentage of the genome covered by each additional topology plateaus; Fig. S1) to exhaustively search for relationships in which subclades or individual Barombi Mbo species were more closely related to riverine populations than other species in the crater lake, suggesting secondary gene flow into this subclade (i. e., introgression) or differential sorting of ancestral polymorphism that originated prior to colonization or after a hybrid swarm. Because we instructed *Saguaro* to propose trees beyond the normal stopping rules based on the percentage of the genome explained by additional topologies to exhaustively search for introgressed regions, the genomic proportions assigned to each tree may not be accurate. These proportions are therefore treated with caution and only used to complement the other results in this study. Details on the *Saguaro* analysis and filtering strategies for calculating proportions are provided in the supplementary methods.

We also characterized differential introgression within subclades of the radiation on both a genome‐wide and local level. We tested for differential introgression between Barombi Mbo species and riverine populations at the genome‐wide level using *f_4_* statistics (Reich et al. 2009; Patterson et al. 2012; Pickrell and Pritchard 2012). We focused on tests of introgression with the three surrounding riverine populations of the most closely related outgroup *Sarotherodon galilaeus* in the nearby Mungo and Meme rivers (MM) and *S. galilaeus* from the more distant Cross River (CR). Based on the tree ((P1, P2),(*S. galilaeus* MM, *S. galilaeus* CR)), *f_4_* statistics were calculated for combinations of species among (a) *Stomatepia*, (b) the *Konia + Pungu* subclade, and (c) *Myaka myaka* with *S. linnelli* as a representative of its sister *Sarotherodon* group. This subset of groupings was chosen to make these analyses more tractable by focusing on species with unique trophic ecologies within the radiation.

Genome‐wide *f_4_* statistics were calculated using the fourpop function in *TreeMix* (Pickrell and Pritchard 2012). Standard error was estimated by jackknifing in windows of 1000 adjacent SNPs to account for linkage disequilibrium. We also visualized the directionality of genome‐wide introgression detected with the *f_4_* statistics using *TreeMix* (v 1.13) (Pickrell and Pritchard 2012). *TreeMix* estimates a maximum likelihood phylogeny of the focal populations and then fits a user‐specified number of migration edges to the tree by comparing genetic covariances among populations. We ran *TreeMix* with *S. galilaeus* as root, and with 0 through 20 migration edges. To determine the most likely number of migration events, we performed likelihood‐ratio tests comparing each graph to one fewer migration event, starting with 1 versus 0 events, and took the first nonsignificant comparison as the most likely number of migration events.

We investigated whether observed signatures of differential introgression at the genome‐wide level contributed variation potentially important to the divergence between species using the *f_d_* statistic, a test statistic similar to *f_4,_* but designed to be calculated across sliding genomic windows (Martin et al. 2015b). The *f_d_* statistic, a modified version of the *D*‐statistic, looks at allele frequencies fitting two allelic patterns referred to as ABBA and BABA based on the tree ((P1,P2),P3,O)), where O is an outgroup species in which no gene flow is thought to occur with the other populations (Martin et al. 2015b). We used individuals of *Coptodon kottae* from another Cameroon crater lake (Fig. 1A) as our distantly related outgroup population for this test and focused on introgression between Barombi Mbo species and surrounding riverine populations *S. galilaeus* MM and *S. galilaeus* CR. Based on the tree ((P1,P2),(*S. galilaeus*, *C. kottae*)), the *f_d_* statistic was calculated for the same combinations of populations used in the genome‐wide tests in 50‐kb sliding windows with a minimum of 100 variant sites with no missing data within a population using the ABBABABA.py script (Martin et al. 2015b, available on https://github.com/simonhmartin/genomics_general). Significance of *f_d_* values in sliding windows across the genome was evaluated using simulations with no migration using *ms‐move* (Garrigan and Geneva 2014). We used the multi‐species Markovian coalescent approach (MSMC: (Schiffels and Durbin 2014)) to estimate effective population size changes through time in the Barombi Mbo cichlids and riverine outgroups to parameterize our simulations. Candidate regions were those containing *f_d_* values above the values calculated from simulated windows. For more details on the sliding window calculations of *f_d_* and significance assessment, see supplementary methods.

We divided candidate introgressed regions into three categories: (1) regions found only in a single Barombi Mbo species, (2) regions shared with another species within the same subclade (suggestive of introgression before divergence of the subclade), and (3) regions shared between two or more species across the entire clade, suggesting sympatric speciation after a hybrid swarm (i.e., differential sorting of ancestral polymorphism) or secondary gene flow into multiple subclades (i.e., introgression). We also looked for overlap between introgressed candidate regions between tests involving *S. galilaeus* MM and *S. galilaeus* CR, another pattern suggestive of a hybrid swarm after initial colonization. For each of these regions, we looked for annotated genes using the well annotated NCBI *Oreochromis* Annotation Release 102 and searched their gene ontology in the phenotype database *Phenoscape* (Mabee et al. 2012; Midford et al. 2013; Manda et al. 2015; Edmunds et al. 2016) and *AmiGO2* (Balsa‐Canto et al. 2016) for functions related to the trophic specializations and observed morphological differences among specialist species, such as skeletal system, circulatory system, metabolism, or pigmentation. It is possible that some of the topologies consistent with introgression with outgroups and introgressed regions from *f_d_* tests stem from introgression from unsampled or extinct populations rather than *S. galilaeus* MM or *S.galilaeus* CR directly; however, this should not change the overall conclusion that secondary gene flow events occurred in the history of the radiation or the functional support for the importance of the introgressed regions that we detected.

### COMPARISON OF PATTERNS OF INTROGRESSION TO PATTERNS OF GENETIC DIVERGENCE AND DIVERSITY

Reduced levels of genetic polymorphism in a population may indicate a strong selective sweep. We examined introgressed regions found in only a single Barombi Mbo species for evidence of selection, suggesting that secondary gene flow brought in variation potentially important for speciation. To examine genetic diversity in candidate introgressed regions, we calculated between‐population nucleotide divergence (*D_xy_*) and within‐population nucleotide diversity (π) for pairwise species comparisons among our Barombi Mbo focal specialist species and the riverine outgroups over the same 50‐kb windows as the *f_d_* tests (see supplementary methods for more details on these calculations).

## Results

### WIDESPREAD EVIDENCE OF POLYPHYLY IN BAROMBI MBO RADIATION SCATTERED ACROSS SMALL REGIONS OF THE GENOME

After conservative filtering of segments to remove uninformative regions (Table S1), the Barombi Mbo cichlid radiation formed a monophyletic group across 53% of the genome whereas only 0.6% was assigned to phylogenies indicating a polyphyletic Barombi Mbo using the machine‐learning *Saguaro* approach. These polyphyletic relationships are consistent with many processes, including secondary gene flow, incomplete lineage sorting, strong selection (balancing, divergent, background), and ancestral population structure. The most prevalent phylogeny spanned 38.2% of the genome and featured the expected species phylogeny for this group, in which all Barombi Mbo individuals formed a single clade with distant relationships to outgroup riverine *S. galilaeus* populations (Fig. 1B). The second most prevalent topology (spanning 11.8% of the genome) featured identical evolutionary relationships, except for a much shorter branch leading to *S. galilaeus* Mungo and Meme River. Branch lengths produced by *Saguaro* have no direct interpretation as an evolutionary distance (analogous to a neighbor‐joining tree), but may be useful for comparison to similar topologies with different branch lengths, Change to (e.g., indicating regions with higher divergence rates; Zamani et al. 2013).

**Figure 2:**
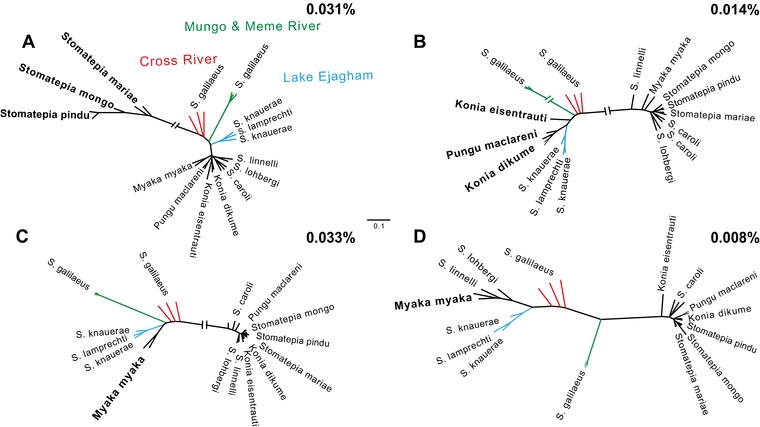
**Topologies featuring Barombi Mbo polyphyly with riverine populations involving the Barombi Mbo species with unique ecologies**. Across small and independent proportions of the genome (A) the entire *Stomatepia* clade, (B) only *S. pindu*, (C) *M. myaka* and *Sarotherodon* species, and (D) only *M. myaka* were more closely related to outgroups than other Barombi Mbo species. These topologies are consistent with introgression between outgroups and Barombi Mbo and are supported by maximum likelihood analyses. Percentages indicate proportion of the genome assigned to these topologies.

In the 0.6% of the genome supporting polyphyletic Barombi Mbo relationships, we found evidence consistent with multiple colonizations of the lake. Since we were looking for patterns consistent with secondary gene flow or a hybrid swarm for subclades of the radiation, we focused on topologies where single species or entire subclades were more closely related to outgroups than other Barombi Mbo species, which represented only 0.24% of the genome (Fig. 2; Figs. S2–S4). Some topologies featured an entire subclade (e.g., *Stomatepia*) as a monophyletic group more closely related to the riverine populations than other Barombi Mbo species, a relationship consistent with a hybrid swarm scenario before the diversification of the *Stomatepia* subclade (Fig. 2A–C). Other topologies featured individual species more closely related to outgroup riverine populations than sister species (Fig. 2D), a relationship consistent with secondary gene flow into that lineage after the initiation of divergence.

The general pattern of polyphyletic relationships was also found in maximum likelihood phylogenetic analyses of a sample of polyphyletic regions initially estimated using *Saguaro*. However, nearly all *Saguaro* topologies in which a single species was more closely related to outgroups differed from the maximum likelihood tree of the same region, which instead featured several Barombi Mbo species as more closely related to the outgroups. The topologies that were consistent across the two analyses and had greater than 85% bootstrap support for the polyphyletic relationship featured entire subclades of Barombi Mbo as more closely related to riverine populations (Fig. 2). For example, in *Stomatepia* we found topologies that grouped multiple species with riverine populations (Fig. 2A). In the *Konia* + *Pungu* subclade, we saw a similar pattern in a topology in which the entire subclade was sister to the riverine outgroup populations (Fig. 2B). In the zooplanktivore *M. myaka*, we did find a topology in which *M. myaka* was sister to the riverine populations (Fig. 2C), but also a topology where *M. myaka*, along with all the Barombi Mbo *Sarotherodon* species, were sister to the riverine outgroup populations (Fig. 2D).

### GENOME‐WIDE EVIDENCE FOR DIFFERENTIAL INTROGRESSION INTO THE RADIATION

Consistent with evidence of differential introgression from RADseq data (Martin et al. 2015a), genome‐wide *f_4_* tests provided evidence of genome‐wide differential gene flow between some Barombi Mbo sister species and the outgroup riverine species (Table 1). There was significant evidence of introgression in genome‐wide tests in tests involving both *S. pindu* in the *Stomatepia* species complex and the hypoxia specialist *K. dikume* in the *Konia* + *Pungu* subclade. Some species pair combinations within these subclades did not show evidence of differential gene flow, suggesting that sympatric speciation potentially occurred for at least some species within the radiation. For example, there was no significant secondary gene flow detected genome‐wide in the tests involving sister species *S. mariae* and *S. mongo* or *M. myaka* and *S. linnelli* (Table 1). These results are similar to previous RADseq genome‐wide *f_4_* statistics of the radiation, with significant secondary gene flow detected into Barombi Mbo from tests involving *S. pindu* and *S. linnelli* (Martin et al. 2015a). In the previous study, these tests were only significant when more closely related *Sarotherodon* species from Lake Ejagham were used as outgroups rather than more distantly related riverine populations of *S. galilaeus* from Ghana and the Democratic Republic of Congo. It is possible that the introgression detected previously came from an unsampled or extinct riverine population; more likely, there was introgression from the Cameroon *S. galilaeus* riverine populations examined in this study into the Ejagham *Sarotherodon* species examined previously (Martin et al. 2015a).

**Table 1:** **Genome‐wide *f_4_* statistics supporting differential introgression within Barombi Mbo radiation**. Tests with significant evidence for differential introgression are highlighted in bold. The *f_4_* statistic was calculated for pairwise combinations among sister species of Barombi Mbo subclades (A, B) and riverine populations of *S. galilaeus* from the Mungo and Meme Rivers (MM) and Cross River (CR)

Introgression with riverine outgroups: (A,B) ← → (*MM, CR*)	*f_4_* statistic	*Z‐*score	*P‐*value
*S. mariae, S. mongo*	−2.04 × 10^−7^ ± −5.15 × 10^−7^	−0.39	0.69
***S. mariae, S. pindu***	−**1.92 × 10^−6^ ±** −**4.48 × 10^−7^**	−**4.29**	**1.8 × 10^−5^**
***S. mongo, S. pindu***	−**1.59 × 10^−6^ ±** −**4.98 × 10^−7^**	−**3.19**	**0.0014**
***K. dikume, K. eisentrauti***	−**2.4 × 10^−6^ ±** −**6 × 10^−7^**	−**4.01**	**6.3 × 10^−5^**
*K. eisentrauti, P. maclareni*	−2.12 × 10^−7^ ± −6.15 × 10^−7^	0.35	0.73
***K. dikume, P. maclareni***	−**2.56 × 10^−6^ ±** −**5.86 × 10^−7^**	−**4.37**	**1.2 × 10^−5^**
*M. myaka, S. linnelli*	−4.04 × 10^−7^ ± −7.11 × 10^−7^	0.56	0.57

**Figure 3:**
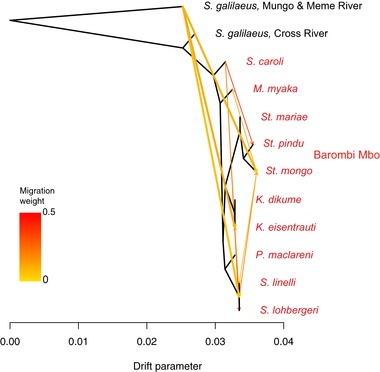
**Visualization of genome‐wide introgression from riverine *Sarotherodon* populations into Barombi Mbo radiation**. *TreeMix* graph illustrating 10 admixture events (with heat colors indicating intensity) on a population graph of the radiation. Admixture events from riverine populations into the radiation are indicated with thicker arrows.

We also found evidence for widespread gene flow connecting populations across Barombi Mbo and neighboring riverine populations in highly interconnected population graphs using *TreeMix* (Pickrell and Pritchard 2012); the likelihood of each graph did not plateau until reaching 10 admixture events (Fig. 3). On the *TreeMix* population graph with 10 admixture events, gene flow from the Mungo/Meme River populations of *S. galilaeus* occurred directly into individual species *S. mongo* and *K. eisentrauti* rather than the ancestral node of their respective subclades (Fig. S5). The proportion of admixture inferred for these two events (0.1% into *S. mongo* and 0.4% into *K. eisentrauti*) was similar to the small proportions of the genome assigned to topologies consistent with secondary gene flow in the *Saguaro* analyses. These admixture events pointing to the tips of the graphs suggest secondary gene flow between nearby riverine populations and individual species within the radiation. In all population graphs allowing up to 21 migration events, any admixture from outgroup riverine populations appears to be coming from the Mungo and Meme rivers rather than the Cross River, consistent with the closer geographic proximity of the former drainages.

### VERY FEW GENOMIC REGIONS CONTAIN SIGNATURES OF DIFFERENTIAL INTROGRESSION INTO INDIVIDUAL BAROMBI MBO SPECIES

**Figure 4:**
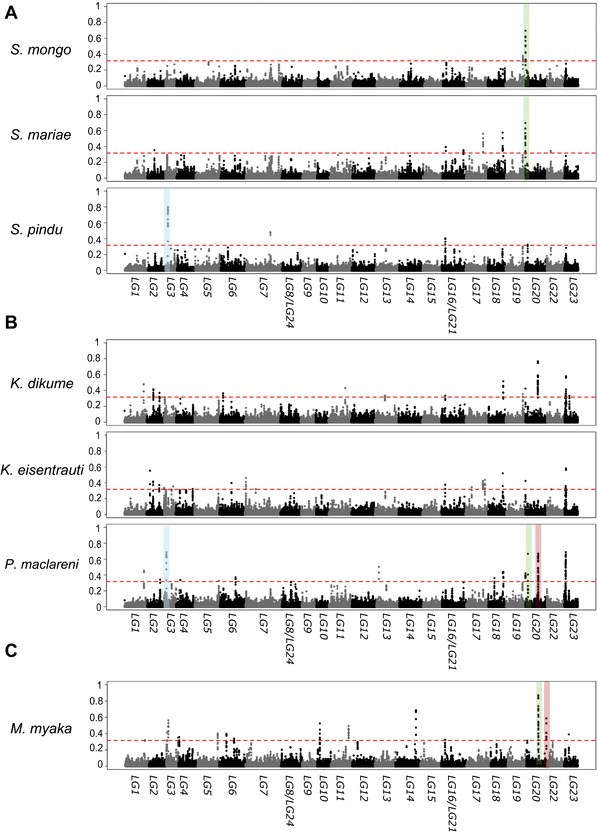
**Manhattan plots of *f_d_* values between riverine populations of *S. galilaeus* from Mungo and Meme river and (A) the *Stomatepia* species, (B) combinations of the three species in *Konia* and *Pungu*, and (C) *Myaka myaka***. Alternating gray/black colors indicate different linkage groups. Dotted red lines mark the coalescent simulation‐based significance thresholds for each test (*f_d_* = 0.315). Peaks highlighted in colors represent those signals of introgression shared across different subclades. Manhattan plots for the scaffolds not assigned to the 24 linkage groups are presented in Figure S8.

We characterized heterogeneity in introgression across the genome by calculating *f_d_* statistics in 50‐kb sliding windows among the same combinations of Barombi Mbo species used in genome‐wide tests to investigate whether differential introgression contributed variation potentially important in the divergence between species. Very few regions of the genome introgressed into single species from *S. galilaeus* MM (Fig. 4A–C; Fig. S6). Among *Stomatepia* species, only a single candidate introgressed region was found to be differentially introgressed from *S. galilaeus* MM into *S. pindu*, *S. mariae*, and *S.mongo* (50, 80, and 80‐kb, respectively), suggesting very little secondary gene flow after initial diversification of *Stomatepia* began (Table 2). Similarly, secondary introgression was also detected for both *Konia* species, *P. maclareni* and *M. myaka* (Table 2). However, only 0.0054–0.1% of the genome introgressed into a single species of a Barombi Mbo subclade from *S.galilaeus* MM, in blocks ranging from 50 to 95‐kb in size. Most of the introgressed regions determined from *f_d_* statistics overlapped with regions assigned to introgression topologies from *Saguaro* (Fig. 2), although these topologies made up only a proportion of each of the introgressed regions (0.09–43.7%; Table S2). This incomplete overlap may be driven by the fixed window size of the *f_d_* statistic test, or the exhaustive genome partitioning in our *Saguaro* approach leading to oversegmentation of the topologies in these regions.

**Table 2:** **Candidate introgressed regions in Barombi Mbo cichlid radiation**. These regions feature significant *f_d_* values between riverine populations of *S. galilaeus* (MM: Mungo and Meme River; CR: Cross River) and the three subclades of the radiation focused on in this study. Unannotated regions with no GO terms are marked with (–)

Linkage Group	Position	Gene(s)	Gene Ontology Terms
***Myaka myaka***			
**LG3**	17560001–17615000	***fcgr2b;fcgr1***	IgG binding, immunoglobin mediated immune response; IgG binding, phagocytosis
**LG4**	800001–855000	***ifi44***	defense response to virus; GTP binding
**LG5**	35530001–35595000	***matn4;rbpjl***	growth plate cartilage chondrocyte morphogenesis; transcriptional activitor activity, RNA polymerase II proximal promotor
**LG6**	10670001–10740000	***prf1;actb***	immunological synapse formation, wide porin channel activity; dense body, focal adhesion
**LG6**	20935001–21025000	***jpt2***	cytosol; plasma membrane
**LG11**	28280001–28375000	***iqgap3;ttc24;gnrh2;igdcc3;polr3gl;hfe***	calmodulin binding, Ras protein signal transduction; biological process; gonadotropin hormone‐releasing activity, reproduction; neuromuscular process controlling balance; nuclear chromatin DNA‐directed RNA polymerase; iron ion transport
**LG20**	1705001–1755000	***atp6ap1;taz***	Rab GTPase binding; positive regulation of bone resorption; O‐acyltransferase activity
**LG20**	19570001–19630000	***sbk1***	protein serine/threonine kinase activity
**NT_167475.1**	450001–500000	***ier5l;h2dmb1;plgrkt***	biological process; ribonuclease H2 complex; positive regulation of plasminogen activation
**NT_167500.1**	930001–1000000	***cldn15; il20rb***	cell‐cell junction assembly, transforming growth factor beta receptor signaling pathway; interleukin‐ 10 receptor activity
**NT_167568.1**	10001–60000	***zfp235***	DNA binding, transcription activity
**NT_167617.1**	320001–400000	***zg57;nebl***	unknown; cardiac muscle thin filament assembly
**NT_167716.1**	220001–270000	**–**	–
**NT_167790.1**	255001–270000	**–**	–
**NT_167636.1**	140001–190000	–	–
***Pungu maclareni***			
**LG3**	14525001–14575000	***plac8***	negative regulation of apoptotic process, chromatin binding
**LG17**	9260001–9315000	***gimap8***	GTP binding, regulation of T cell apoptotic process
**NT_167557.1**	1410001–1495000	***pgbd4*;UPC**	DNA binding transcription factor activity
**NT_167671.1**	265001–330000	***wsc3*; UPC(2)**	Rho protein signal transduction
**NT_167747.1**	45001–330000	***nlrc3;dclk2***	negative regulation of I‐kappaB kinase/NF‐kappaB signaling; peptidyl‐serine phosphorylation
**NT_168010.1**	30001–80000	–	–
		***Konia dikume***	
**LG2**	18660001–18710000	***hmcn1***	basement membrane; fin morphogenesis
**NT_168013.1**	1–70000	***siglec12;* UPC(2)**	cell adhesion, carbohydrate binding;
***Konia eisentrauti***			
**LG6**	22460001–22515000	***pitpnc1; vwa7***	phosphatidylinositol transporter activity; extracellular region, biological process
**NT_167586.1**	220001–290000	***trappc1;psmb6;ephb4*** *	TRAPP complex; threonine‐type endopeptidase activity; ephrin receptor signaling pathway, heart morphogenesis
**NT_167663.1**	535001–630000	***s100p;s100g; slc29a2*; UPC**	RAGE receptor binding; apical plasma membrane, transition ion binding; nucleoside transmembrane transporter activity;‐
***Stomatepia mongo***			
**LG20**	150001–230000	***plod3;cyb5d;clec10A***	peptidyl‐lysine hydroxylation, procollagen‐lysine 5‐dioxygenase activity; metal ion binding; carbohydrate binding, adaptive immune response
***Stomatepia mariae***			
**LG16‐21**	31140001–31220000	***cxcr2;faim; parp9;parp14***	nuetrophil chemotaxis; apoptotic process; positive regulation of interferon‐gamma‐mediated signaling pathway; negative regulation of interferon‐gamma‐mediated signaling pathway
***Stomatepia pindu***			
**NT_167675.1**	605001–655000	***cmklr1;c5ar1***	G‐protein coupled receptor signaling pathway, chemotaxis; complement component C5a receptor activity

*best candidate region for secondary gene flow contributing to diversification; UPC = uncharacterized protein coding gene

In contrast, a larger proportion of the genome appears introgressed from *S. galilaeus* CR based on our coalescent simulation cut‐offs (Fig. S7). Introgression with *S. galilaeus* CR and a single species of Barombi Mbo was detected in 0.7–5% of the genome. These candidate introgressed regions ranged in size from 50 to 220‐kb. The size range of introgressed regions was larger in tests with *S. galilaeus* CR than *S. galilaeus* MM (Fig. S8). Larger blocks of introgressed regions could indicate that gene flow from Cross River was more recent than Mungo or Meme River. Absolute genetic divergence between Barombi Mbo species and *S. galilaeus* CR is about seven times smaller across the genome than between Barombi Mbo and *S. galilaeus* MM (Fig. S9–S11; Table S3). It is difficult to assess whether all these candidate introgressed regions from *S. galilaeus* CR represent long tracts of introgressed material given that (1) our moderate sequencing depth prevents phasing and (2) the percentage and length of introgression tracts may change based on the timing of gene flow and divergence in this system. Such knowledge will require demographic modeling of different scenarios of gene flow and its timing, which is still computationally intractable for 11 species. Given this, we focus on the candidate introgressed regions involving *S. galilaeus* MM population. We also use the *S. galilaeus* CR introgressed regions that overlap with the *S. galilaeus* MM introgressed regions to look for patterns of secondary gene flow consistent with a hybrid swarm.

### EVIDENCE FOR A HYBRID SWARM

We found shared introgression signals between species both within and among subclades of Barombi Mbo (Fig. 3, Table S4 and S5) in which multiple species shared introgressed regions from a riverine population. This suggests that some of this introgression may have occurred in the ancestral stages of the radiation and differentially sorted among species as they diversified. A majority of these (19/27) were also candidate introgression regions from *S. galilaeus* CR, in which one or more Barombi Mbo species are more closely related to *S. galilaeus* MM while other Barombi Mbo species are more closely related to *S. galilaeus* CR (Table 2). This pattern is suggestive of a hybrid swarm scenario from multiple colonizations by different riverine populations before diversification and the sorting of these ancestral polymorphisms among incipient Barombi Mbo species.

### CANDIDATE GENES FOR ADAPTIVE DIVERSIFICATION WITHIN INTROGRESSED REGIONS

Although we found evidence of differential introgression among sister species scattered across a small proportion of the genome, the types of genes found in these regions do not paint a clear picture of how this variation may have contributed to speciation (Table 2). For example, differential introgression in *Stomatepia* occurred in regions with genes involved in immune response (Table 2), with no obvious links to the divergent morphological, ecological, or color patterning traits observed among these species (Martin 2012) nor to those traits normally associated with adaptive radiation in cichlid fishes such as body shape, pharyngeal jaw morphology, retinal pigments, or male coloration (Kocher 2004; Barluenga et al. 2006; Wagner et al. 2012; Brawand et al. 2014; Malinsky et al. 2015; Meier et al. 2017). Similarly, in both the *Konia* + *Pungu* and *Myaka* + *Sarotherodon* subclades, introgressed regions were near genes involved in a large range of biological processes not directly associated with adaptive ecological traits in these species, such as *K. dikume*’s hypoxia tolerance, *P. maclareni's* spongivory, and *M. myaka's* zooplanktivory.

**Figure 5:**
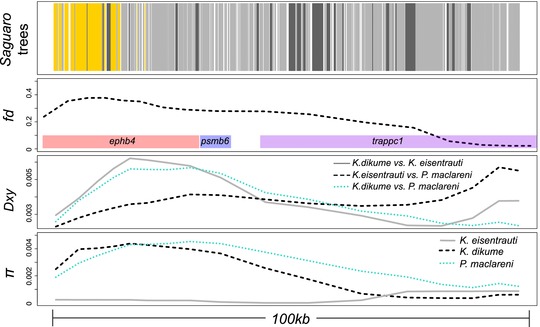
**Candidate adaptive introgression region in the *Konia* species pair containing gene *ephb4***. Row 1 shows the topologies assigned by *Saguaro* to the region. Gold blocks were assigned to a topology featuring the *Konia* species pair plus *Pungu maclareni* sister to the riverine *S. galilaeus* populations (Fig. 2B), light grey blocks were assigned to the predominant monophyletic topology (Fig. 1B), and dark grey blocks were assigned to any other topology. Row 2 shows the peak signal of introgression across scaffold NT_167586.1 detected from the *f*_d_ statistic across the three‐test combination involving *K. dikume*, *K. eisentrauti*, *Pungu maclareni* and riverine populations of *S. galilaeus* MM in overlapping 50‐kb windows and the genes in this peak (*ephb4, psmb6*). Row 3 shows between‐population divergence (*D_xy_*) among the combinations of *Konia* and *Pungu* species calculated in overlapping 50‐kb windows. Row 4 shows within‐population diversity (*π*) in the same non‐overlapping 50‐kb windows for the species of *Konia* and *Pungu*. The data from rows highly 2–4 were smoothed using the function smooth.spline in R with a spar of 0.1 for ease of visualization in the figure.

The clearest candidate for adaptive diversification showing differential introgression into a single species was found between the shallow‐water detritivore *K. eisentrauti* and deep‐water hypoxia specialist *K. dikume* (Fig. 5). One introgressed region in *K. eisentrauti* contains *ephb4*, a gene involved in blood vessel formation (Herbert et al. 2009; Kawasaki et al. 2014). *K. dikume's* deep water specialization includes higher blood volume with higher concentrations of hemoglobin (Green et al. 1973). This region overlapped with a region of the genome assigned to a polyphyletic Barombi Mbo topology that featured both *Konia* species plus *P. maclareni* sister to riverine outgroups by *Saguaro* (Fig. 2B, 5). However, this candidate introgressed region is found within a small scaffold that predominantly exhibits *f_d_* values slightly above our simulation cutoff. False positive *f_d_* values can arise from low nucleotide diversity in a region (Martin et al. 2015b), so even this seemingly convincing candidate gene introgression may be driven by lower genetic diversity in the *K. eisentrauti* sequence compared to *K. dikume* rather than true introgression with *S. galileaus* MM.

From the perspective of gene annotations, there is better support for the contributions of secondary gene flow to the radiation in the introgressed regions that were shared across multiple species of the radiation (Table S4–S5). For example, a region introgressed into *K. eisentrauti* and *P. maclareni* contains *pafah1b3*, a gene involved in platelet activation activity and spermatogenesis in mice (Prescott et al. 2000; Koizumi et al. 2003; Yan et al. 2003), which while not directly associated with *K. dikume's* traits of higher blood volume with higher concentrations of hemoglobin, is still associated with blood (Table S4). Another region contains an olfactory receptor gene, *or52e8*. The introgressed region containing *or52e8* has signatures of introgression from *S. galilaeus* MM with *K. dikume* and *M. myaka*, as well as from *S. galilaeus* CR with *K. eisentrauti*, *S. mongo*, and *S. mariae* (Table S5). Olfactory signaling is a key component of mate choice and species recognition in cichlids (Plenderleith et al. 2005; Blais et al. 2009). For instance, introduction of olfactory sensory variation from riverine populations may have been important for the divergence of *S. mariae* and *S. pindu*, which appear to exhibit greater sexual than ecological divergence (Martin 2012). Similarly, introgression of olfactory alleles may have played a key role in triggering rapid speciation in Lake Ejagham *Coptodon* cichlids after an 8000 year waiting period (Poelstra et al. 2018).

Finally, two shared introgressed regions contained genes with known skeletal effects: *pfn1* and *mdn1*. The first has been shown to effect skeletal development of long bones of limbs in mice (Böttcher et al. 2009) and cell migration during gastrulation in zebrafish (Lai et al. 2008). Mutations in *mdn1* have known craniofacial effects in zebrafish, causing changes in cranium, mandibular arch, and eye size (Kettleborough et al. 2013). The region containing *mdn1* has signatures of introgression from *S. galilaeus* MM with *P. maclareni* and *S. pindu*, as well as introgression from *S. galilaeus* CR with *K. dikume* and *S. mongo*. Craniofacial traits such as jaw length, head depth, and orbit diameter are divergent among these Cameroon cichlid species (Martin 2012). For instance, *P. maclareni* has smaller and thicker oral jaws, variation in tooth size, and thicker lips compared to other Barombi Mbo species (Trewavas et al. 1972). *Stomatepia mongo* has a highly elongated neurocranium compared to sister species *S. mariae* and *pindu* (Trewavas et al. 1972; Musilová et al. 2014). Signatures of introgression from different riverine populations in regions containing putative adaptive alleles suggest that secondary gene flow from multiple riverine populations prior to diversification (i.e., a hybrid swarm scenario) may have contributed variation important for the radiation.

### WEAK SUPPORT FOR SELECTION ON CANDIDATE INTROGRESSED REGIONS

Within the limitations of our sample sizes for each population in this study, the amount of genetic diversity in introgressed regions does not suggest strong divergent selection on introgressed genetic variation due to selective sweeps. In line with the presence of peaks in *f_d_* values in these regions, between‐population diversity (*D_xy_*) was typically high between one of the species and its sister species (Fig. 4; Table S6). However, within‐population diversity across many of these regions was often greater or comparable to scaffold and genome‐wide averages (Table S6), suggesting these regions may not have experienced hard selective sweeps that would support their role in adaptive divergence among species. Only a few differentially introgressed regions in *S. pindu* (*n* = 1), *S. mongo* (*n* = 1), and *P. maclareni* (*n* = 1) exhibited genetic diversity an order of magnitude lower than the linkage group average (Table S6), consistent with a selective sweep.

In summary, although we found evidence for differential secondary gene flow between sister species in the radiation, we did not find clear evidence from introgressed genes with well‐known roles in adaptive divergence between cichlids nor strong evidence of selection on these regions. However, these alleles may certainly still serve adaptive functions or may be involved in genetic incompatibilities between species. Furthermore, given our sample size limitations, we cannot yet provide a good estimate of selection strength on these regions.

## Discussion

### EQUIVOCAL EVIDENCE THAT SECONDARY GENE FLOW PROMOTED THE DIVERSIFICATION OF BAROMBI MBO CICHLIDS

Our fine‐scale investigations of introgression across the genomes of a celebrated putative example of sympatric speciation are consistent with two possible scenarios: (1) sympatric speciation in the presence of continuous neutral secondary gene flow into the radiation, or (2) speciation initiated by secondary gene flow; our data are not consistent with a scenario involving an extensive period of allopatry and secondary contact promoting speciation. We found little support for the latter allopatric scenario using both *Saguaro* machine learning and sliding‐window *f_d_* statistics to exhaustively search for differential introgression into single species. From the *Saguaro* analyses, our most conservative estimate of introgression into a single species within the radiation ranged from 0.013–0.019% of the genome. Estimates are similarly small for the *f_d_* statistics, ranging from 0.0054–0.1% of the genome from *S. galilaeus* populations in the neighboring Mungo and Meme Rivers. Furthermore, even these significant outliers may represent false positives, particularly in the case of introgression with *S. galilaeus* from Cross River, where 0.7–5% of the genome appears to have introgressed from this population. More complex coalescent simulations, larger sample sizes, and knowledge of the timing of diversification and gene flow events are needed to accurately assess the signatures observed. It is also difficult to distinguish signatures of differential introgression from the biased assortment of ancestral polymorphism into modern lineages; (i.e., a hybrid swarm scenario that is still consistent with sympatric divergence entirely within the crater lake). Finally, even if our statistical outliers represent differentially introgressed regions, their importance to the speciation process is equivocal. Within the limitations of our sample sizes, we found no evidence of selective sweeps in most of these regions that would suggest they aided in divergence between species and most regions contained housekeeping genes that do not clearly suggest how introgressed variation would have contributed to the radiation. Nonetheless, these genes may still serve an adaptive function or could be involved in genetic incompatibilities between diverging sympatric populations, possibly introduced by an initial hybrid swarm in the lake (Seehausen 2004, 2013; Schumer et al. 2015).

### EVIDENCE FOR A HYBRID SWARM FURTHER COMPLICATES THE ROLE OF GENE FLOW IN THE SPECIATION PROCESS

Beyond speciation scenarios involving secondary gene flow aiding the completion of speciation, our findings are suggestive of another scenario for sympatric speciation in this system: sympatric speciation from an initial hybrid swarm resulting from the differential sorting of ancestral polymorphisms among incipient species (e.g., see Fig. 1 in Martin et al. 2015a). A hybrid swarm is not easily detectable using the *f_d_* statistic because introgressed variation could be shared among diverging sister species, producing an *f_d_* value near zero due to a BBBA rather than ABBA pattern (Reich et al. 2009; Patterson et al. 2012). However, some of the *f_d_* peaks appear to be (1) shared across at least two of the sister species in a subclade, (2) shared between species of different subclades, or (3) contain variation from both riverine populations (Mungo/Meme and Cross Rivers) that appears differentially sorted among sister species. All three of these patterns are consistent with an ancestral hybrid swarm before divergence between sister species occurred. This pattern of differential sorting of variation from a hybrid swarm from *f_d_* analyses could also result from a lack of power in this statistic to distinguish the directionality of the introgression detected in those regions when using biallelic patterns and four populations (e.g., when two populations share similar allele patterns, the other two populations can share the opposite allele pattern by default). However, we also found evidence that entire subclades (e.g., *Stomatepia*) were more closely related to riverine populations than other Barombi Mbo subclades using our *Saguaro* analyses, a pattern that is also consistent with a hybrid swarm (e.g., Fig. 2).

It is in these regions that we find the best candidate genes for secondary gene flow contributing to diversification in this system. These candidates are known to affect important traits in other adaptive radiations of cichlids, including olfactory signaling (Nikaido et al. 2013, 2014; Azzouzi et al. 2014; Keller‐Costa et al. 2015) and pharyngeal jaw morphology (Muschick et al. 2011; Brawand et al. 2014; Malinsky et al. 2015). These findings are similar to studies on other systems using similar approaches which found compelling cases for adaptive introgression contributing to diversification (e.g., Abi‐Rached et al. 2011; The Heliconius Genome Consortium et al. 2012; Huerta‐Sánchez et al. 2014; Lamichhaney et al. 2015; Stankowski and Streisfeld 2015; Arnold et al. 2016; Meier et al. 2017), including our own previous work (Richards and Martin 2017). For example, several studies have found convincing candidate genes/variants in introgressed regions suggesting that adaptive introgression played a role in shaping ecological and morphological diversity. These include the detection of introgressed alleles linked to wing‐color patterning involved in mimicry and mate selection in *Heliconius* butterflies (The Heliconius Genome Consortium et al. 2012), flower coloration involved in pollinator preferences for *Mimulus* species (Stankowski and Streisfeld 2015), and oral jaw size variation involved in scale‐eating trophic specialization in *Cyprinodon* pupfishes (Richards and Martin 2017).

### THE CHALLENGES OF SUPPORTING OR REJECTING A ROLE FOR SECONDARY GENE FLOW FROM TESTS OF DIFFERENTIAL INTROGRESSION

There are some caveats to our interpretations of secondary gene flow and its functional role in the ecological and morphological diversity observed within the lake. Recombination rate varies across genomes and determines the scale over which patterns of admixture and differentiation vary (Smukowski and Noor 2011). In our fixed sliding window size of 50‐kb, we may have missed important patterns of introgression in regions of recombination hotspots, where such patterns are expected to be very localized (Schumer et al. 2018). Shared variation among species may reflect unsorted polymorphisms from structured ancestral populations rather than hybridization. Introgression events can also be hard to distinguish from ongoing balancing selection of ancestral polymorphism that is sieved between species (Guerrero and Hahn 2017).

Furthermore, although we focused on searching for genetic signatures of hard selective sweeps, introgressed regions with intermediate to high nucleotide diversity may have undergone soft selective sweeps, in which selection drives multiple adaptive haplotypes to fixation. Some of these introgressed regions may have been adaptive and undergone soft selective sweeps, although the relative contributions of hard sweeps versus soft sweeps during adaptation and speciation is still the subject of much debate (Hermisson and Pennings 2005, 2017; Pritchard et al. 2010; Jensen 2014; Schrider et al. 2015).

Although a search for candidate genes with plausible roles in speciation is a good starting point when considering whether introgressed regions contributed to divergence, there are some caveats to ruling out the importance of secondary gene flow based solely on gene annotations. Not all the effects of a gene may be known, even for model organisms, and there is growing support for an omnigenic model of the link between genotype and phenotype, where all genes expressed in relevant cells can potentially influence a trait (Boyle et al. 2017). Likewise, not all traits involved in species divergence are known and more cryptic traits (e.g., metabolism, physiology) could be as important as the more obviously divergent morphological traits (e.g. McGirr and Martin 2018). Understanding which traits and genes are involved in speciation is now becoming the difficult problem of functional genetic analyses for anything sweeping within a population (see also Richards et al. 2018).

Even if there are no coding regions present in the introgressed region, it may still contain important regulatory elements that affect genes in other regions that underlie speciation traits. It could also house variants that cause genetic incompatibilities and provide a source of postzygotic reproductive isolation. Divergence caused by genetic incompatibilities may not leave signatures of hard selective sweeps in the genome either. One prediction of the hybrid swarm hypothesis is that some of the novel allele combinations introduced by hybridization could include segregating genetic incompatibilities (Seehausen 2004, 2013; Abbott et al. 2013). The presence of DMIs has been shown in other African cichlids (Stelkens et al. 2010), but little is known about what specific regions of the genome contribute to these patterns. Detecting candidate regions of DMIs in non‐model organisms is a currently growing body of work mainly investigated in relatively recent hybrid zones (Schumer et al. 2014, 2015, 2018), but is a promising avenue of future research, particularly given the support for a potential hybrid swarm in the evolutionary history of Barombi Mbo shown here.

Although the limitations of this study make it hard to definitively support or reject a role for gene flow in the adaptive radiation of Barombi Mbo, we highlight some of the gaps in knowledge that need to be filled before we can understand what role the observed secondary gene flow played in diversification. A more thorough understanding of the evolutionary history of the group (e.g., the timing and duration of gene flow events, divergence times among species) will be useful in determining the amount of introgression we would expect to see in the contemporary genomes of the species under different speciation scenarios. A thorough investigation of all the regions of the genome that have undergone soft and hard selective sweeps and the timing of those sweeps, alongside a search for genetic incompatibilities among species, will make it easier to understand the functional importance of observed introgressed regions to diversification. The demographic analyses needed for a radiation of 11 species are still largely intractable, but will eventually provide much‐needed insights into the role of the secondary gene flow observed in this classic putative example of sympatric speciation. However, some recently proposed statistics that infer the direction and relative timing of gene flow to speciation provide a promising future avenue for distinguishing between different scenarios of introgression (e.g., hybrid speciation vs. introgression after initial divergence; Hibbins and Hahn 2018) without extensive demographic modeling.

### BEST AND WORST REMAINING CASES FOR SYMPATRIC SPECIATION WITHIN THE BAROMBI Mbo CICHLID RADIATION

Although the radiation as a whole may not have entirely arisen in sympatry, some sister species within Barombi Mbo may be better case studies of the process than others. Within the three‐species *Stomatepia* subclade, there is little evidence that secondary gene flow played an important role in speciation. On a genome‐wide level, we detected secondary gene flow for *S. pindu* and *S. mariae*, but not *S. mongo* (Table 1). This is intriguing given that a previous study found continuous unimodal morphological variation connecting the *pindu/mariae* species complex (Martin 2012), but did not include the rare and morphologically distinctive *S. mongo* (Musilová et al. 2014). Furthermore, while the *pindu/mariae* species complex appears unimodal across ecological phenotypes and trophic axes, it exhibits bimodality along the axis of color pattern, indicating that disruptive sexual selection on patterning may be the primary driver and initial barrier to reproduction for this group (Martin 2012). This provides a category of functional traits to search for when trying to determine if secondary gene flow played a role in their current level of divergence.

No reproductive isolating barriers or genetic incompatibilities have yet been quantified among any Barombi Mbo species (in contrast to the strong assortative mating by size, color, morphology, and diet documented in Lake Ejagham *Coptodon* species (Martin 2013). *S. pindu* and *S. mariae* can produce viable F1 hybrids in a no‐choice laboratory environment (C.H.M. pers. obs.); however, all Barombi Mbo species appear to avoid hybridizing when housed in mixed‐species laboratory aquaria and no hybrid courtship has been observed in the field (C.H.M. pers. obs.). Estimates from scale growth rings suggest only moderate disruptive selection on ecological traits in the *Stomatepia pindu/mariae* species pair. These species were also not significantly different in their dietary source of carbon or relative trophic position from stable isotope analyses (Martin 2012) and their diets are only marginally distinct based on stomach content analysis, containing predominantly insect/shrimp prey in *pindu* and insect/fish prey in *mariae* (Trewavas 1972; C.H.M. unpublished data). Very little is known about reproductive isolating barriers in other Barombi Mbo cichlids, aside from the interesting and unique temporal isolation of *Myaka myaka* due to its seasonal lekking behavior (C.H.M. pers. obs.).

On a fine scale, the few introgressed regions unique to each of the *Stomatepia* species contained only immune response genes. Shared signatures of introgression among two of the three species or with other Barombi Mbo species represented a larger proportion of the genome than differentially introgressed regions within each species, although both types of introgression were rare across the genome. In those regions that introgressed in more than one *Stomatepia* species, but from different riverine populations, we found genes with functions more typically associated with cichlid radiations. Even for the two monotypic specialist species *M. myaka* and *P. maclareni*, introgressed regions found solely in these species contained mainly housekeeping genes, suggesting secondary gene flow may have been neutral in the evolution of their trophic specializations. However, they also share signatures of introgression in the same regions as *Stomatepia*, suggesting that these monotypic specialists may have obtained variation for their specialized traits from the sorting of ancestral polymorphisms within a hybrid swarm before the radiation began.

Among all the ecologically divergent species pairs focused on in this study, *K. eisentrauti* and *K. dikume* are the least convincing as a putative example of sympatric speciation between sister species. Differentially introgressed regions between *K. dikume* and *K. eisentrauti* include a region containing *ephb4*, involved in heart and blood vessel development. Given *K. dikume*’s hypoxia specialization, this region is the best potential candidate in this study for secondary gene flow and an allopatric phase of speciation contributing to diversification between species in the radiation. These two species also exist in microallopatry; *K. eisentrauti* is an abundant detritivore found only along the shallow littoral region of the lake whereas *K. dikume* is a deep‐water specialist on *Chaoborus* midge larvae, which have only been collected in deep‐water gill nets (Trewavas et al. 1972; Schliewen et al. 1994; C.H.M. pers. obs.). Both species are mouthbrooders and likely breed in non‐overlapping habitats although nothing is known about the breeding habits of *K. dikume*.

## Conclusion

The complex history of colonization in the Barombi Mbo crater lake cichlid radiation found in this and a previous genome‐wide study suggests that secondary gene flow may have played a role in the speciation process, which violates one of the strict criteria for demonstrating sympatric speciation in the wild (Coyne and Orr 2004). Our fine‐scale dissection of introgressed regions across the entire genome suggests that supporting or rejecting a role for secondary gene flow in speciation will require an understanding of the functional alleles within each region and their evolutionary history. Nonetheless, we can rule out a scenario in which extensive secondary gene flow after a long allopatric phase, such as reinforcement, contributed to diversification in any Barombi Mbo species. Instead, small and scattered regions of secondary gene flow that were differentially sorted among these sympatric species may have provided variation with undiscovered functional effects on the divergent ecologies and morphologies seen in the lake or this gene flow was predominantly neutral with respect to its role in the speciation process. We found more convincing evidence that secondary gene flow contributed adaptive variation during an initial hybrid swarm within the lake and later sorting of that variation among species in sympatry. Disentangling the effects of a putative hybrid swarm from secondary contact on the speciation process will require a better understanding of the timing of gene flow events compared to the diversification times of Barombi Mbo species. We found evidence for gene flow into the radiation both before and after initial diversification of subclades within the lake. Even without this information, equivocal support for a functional role of secondary gene flow in the radiation of Barombi Mbo cichlids suggests that we should not rule out the possibility of sympatric speciation in this system just yet.

## CONFLICT OF INTEREST

The authors declare no conflict of interest.

Associate Editor: Z. Gompert

## Supporting information


**Table S1. Percentages of the genome assigned to topologies under various filtering criteria**.
**Table S2. Overlap of *Saguaro* and *f_d_* introgressed regions**.
**Table S3. Average *D_xy_* among Barombi Mbo and riverine *Sarotherodon* species across the genome**.
**Table S4. Candidate introgressed regions in Barombi Mbo cichlid radiation that are shared across multiple species in a subclade**.
**Table S5. Candidate introgressed regions in Barombi Mbo cichlid radiation that are shared across multiple species across Barombi Mbo**.
**Table S6. Within‐population genetic diversity in introgressed regions in Barombi Mbo and *Sarotherodon* riverine populations**.

**Figure S1. The percentage of the genome assigned to each topology by *Saguaro***.

**Figure S2. *Saguaro* topologies featuring Barombi Mbo polyphyly with riverine populations involving the *Stomatepia* three‐species complex**.

**Figure S3. *Saguaro* topologies featuring Barombi Mbo polyphyly with riverine populations involving the *Konia + Pungu* subclade**.

**Figure S4. *Saguaro* topologies featuring Barombi Mbo polyphyly with riverine populations involving the *Myaka + Sarotherodon* subclade**.

**Figure S5. The log‐likelihood of *TreeMix* population graphs for Barombi Mbo cichlids as a function of the number of migration events**.

**Figure S6. Visualization of introgression with *S. galilaeus* MM across unassigned scaffolds for Barombi Mbo**.

**Figure S7. Visualization of introgression with *S. galilaeus* CR across linkage groups for Barombi Mbo**.

**Figure S8. Distribution of introgression block sizes detected from sliding window *fd* statistic tests for Barombi Mbo species**.

**Figure S9. Visualization of divergence across the genome for species of *Stomatepia* and *S. galilaeus* populations from Mungo/Meme River (MM) and Cross River (CR)**.

**Figure S10. Visualization of divergence across the genome for species of *Konia* and *S. galilaeus* populations from Mungo/Meme River and Cross River**.

**Figure S11. Visualization of divergence across the genome between *M. myaka* and *S. linnelli*, *S. galilaeus* populations from Mungo/Meme River, and Cross River**.

**Figure S12. Linkage disequilibrium decay among individuals used in this study**.

**Figure S13. Ancestral Population Size of Barombi Mbo Species**.

**Figure S14. Ancestral population size of outgroups lineages**.
