# Supplementary material for: Don't throw out the sympatric speciation with the crater lake water: fine‐scale investigation of introgression provides equivocal support for causal role of secondary gene flow in one of the clearest examples of sympatric speciation
Source: Evol Lett. 2018 Aug 15;2(5):524–40. doi: 10.1002/evl3.78 (PMC6145409; doi:10.1002/evl3.78)
Supplement: Supplementary file 1 — Table S1. Percentages of the genome assigned to topologies under various filtering criteria. Table S2. Overlap of Saguaro and fd introgressed regions. Table S3. Average Dxy among Barombi Mbo and riverine Sarotherodon species across the genome. Table S4. Candidate introgressed regions in Barombi Mbo cichlid radiation that are shared across multiple species in a subclade. Table S5. Candidate introgressed regions in Barombi Mbo cichlid radiation that are shared across multiple species across Barombi Mbo. Table S6. Within‐population genetic diversity in introgressed regions in Barombi Mbo and Sarotherodon riverine populations. [file EVL3-2-524-s001.docx]

Article Type: Letter

**Don’t throw out the sympatric speciation with the crater lake water:**

**fine-scale investigation of introgression provides equivocal support for causal role of secondary gene flow in one of the clearest examples of sympatric speciation**

Emilie J. Richards^1*^, Jelmer W. Poelstra^1,2^, and Christopher H. Martin^1^

^1^ Biology Department, University of North Carolina at Chapel Hill, Chapel Hill, North Carolina, United States of America

^2^ Biology Department, Duke University, Durham, North Carolina, United States of America

*corresponding author: ejr@live.unc.edu

Short title: Role of secondary gene flow in cichlid sympatric speciation

Key words: adaptive radiation, introgression, gene flow, sympatric speciation, population genetics, speciation genomics

**Supplementary Methods**

*Sampling and Genome Sequencing*

We sequenced whole genomes of 1-3 individuals from 10 out of the 11 species within the sympatric radiation of Oreochromini cichlids in Cameroon crater lake Barombi Mbo (excluding *Sarotherodon steinbachi* which is morphologically and ecologically similar to the other three *Sarotherodon* species), an endemic *Sarotherodon* species pair from Lake Ejagham (*S. lamprechti* and *S. knauerae*), and outgroup *Sarotherodon* *galilaeus* individuals from all three river drainages flanking the lake: Cross, Meme, and Mungo rivers (e.g. see map in (Schliewen et al. 1994); Fig 1A). Individual cichlids were caught by seine, gill net, or hook-and-line from Barombi Mbo, Lake Ejagham, Cross River, Mungo River, and Meme River in January, 2010 and July, 2016. Fishes were euthanized in an overdose of buffered MS-222 (Finquel, Inc.) following approved protocols from the University of California, Davis Institutional Animal Care and Use Committee (#17455) and University of North Carolina Animal Care and Use Committee (#15-179.0). Whole specimens or tissue samples were stored in 95-100% ethanol or RNAlater (Ambion, Inc.) in the field.

DNA was extracted from muscle tissue using DNeasy Blood and Tissue kits (Qiagen, Inc.) and quantified on a Qubit 3.0 fluorometer (Thermofisher Scientific, Inc.). Genomic libraries were prepared using the automated Apollo 324 system (WaterGen Biosystems, Inc.) at the Vincent J. Coates Genomic Sequencing Center (QB3) at the University of California, Berkeley. Samples were fragmented using Covaris sonication, barcoded with Illumina indices, and quality checked using a Fragment Analyzer (Advanced Analytical Technologies, Inc.). Nine to twelve samples were pooled in four different libraries for 150PE sequencing on four lanes of an Illumina Hiseq4000.

1.9 billion raw reads were mapped from 28 individuals to the *Oreochromis niloticus* reference genome v1.1 (NCBI, total sequence length = 927,679,487; number of scaffold = 5,909, scaffold N50, = 2,766,223; contig N50 = 29,493) with the Burrows-Wheeler Alignment Tool (BWA-MEM v 0.7.15) (Li and Durbin 2009; Li 2013). Duplicate reads were identified using MarkDuplicates and BAM indices were created using BuildBamIndex in Picard Tools (v 2.10.3, <http://broadinstitute.github.io/picard)>. Average coverage across individuals was 10.39 X (range: 6.19-17.67 X). We followed the best practices guide (Van der Auwera et al. 2013) recommended for the Genome Analysis Toolkit (v 3.5) (DePristo et al. 2011) to call and refine our SNP variant dataset using the program HaplotypeCaller. Because we lacked high-quality known variants for these non-model species, we filtered SNPs based on the recommended hard filter criteria (i.e. QD < 2.0; FS < 60; MQRankSum < -12.5; ReadPosRankSum < -8) (DePristo et al. 2011; Marsden et al. 2014) using vcftools “—remove-filtered-all” flag (v 0.1.14) (Danecek et al. 2011). We further filtered SNPs with a minor allele frequency of less than 0.05, a genotype quality score less than 20, a depth of coverage less than 5, and more than 10% missing data in vcftools.

*Characterization of introgression patterns across the genome with SAGUARO*

First, we exhaustively searched the genomes for patterns of non-monophyletic Barombi Mbo relationships using the machine learning program SAGUARO (Zamani et al. 2013) to identify regions of the genome that contained relationships consistent with expectations from multiple colorizations and secondary gene flow into the radiation (i.e. paraphyletic/polyphyletic Barombi Mbo radiations). Saguaro combines a hidden Markov model with a self-organizing map to characterize variation in phylogenetic relationships among individuals across the genome without requiring *a priori* hypotheses about these relationships or the size of genomic regions. This method infers relationships among individuals in the form of genetic distance matrices and assigns segments across the genomes to different topologies. These genetic distance matrices can then be transformed into neighborhood joining trees to visualize patterns of evolutionary relatedness across the genome. We exhaustively searched the genome for topological variation by partitioning the genome into a total of 75 unique topologies (well past the inflection point at 30 topologies where the percent of genome explained by each additional topology plateaus; Fig S1).

Since smaller segments with fewer informative sites are more likely to be incorrectly assigned to a hypothesized topology by chance, we tested various minimum SNP filters (1, 10, or 20 SNPs) for reducing the amount of short uninformative segments and their effect on the percentage of the genome assigned to topologies. We found that while the percentage of the genome assigned to topologies changes when we apply SNP filters, none of the topologies had all segments entirely removed and the relative proportions of the genome assigned to particular types of topologies were similar across filtering strategies (Table S1). This may be due to uninformative sites being assigned to topologies in a random fashion, such that removing these sites does not change the percentage of the genome disproportionately across topologies. The percentages indicated in the results represent the percentage of base pairs in the genome assigned to topologies after using a 20 SNP minimum filter and represent conservative estimates.

We searched these 75 topologies for evidence of relationships where subclades or individual Barombi Mbo species were more closely related to riverine populations than other species in the crater lake, suggesting sympatric speciation after a hybrid swarm (i.e. differential sorting of ancestral polymorphism) or secondary gene flow into this subclade (introgression). For each of these topologies, we checked if they matched the maximum likelihood topology generated using RaxML from an alignment of the region assigned to the topology by *Saguaro* that contained the most SNPs and a GTRGAMMA model of sequence evolution. Topologies which were not consistent with the maximum likelihood trees with more than 85% bootstrap support were removed from further comparisons to results from other approaches used in this study (e.g. overlap with sliding window tests for introgression).

*Characterization of introgression patterns across the genome using sliding windows of fd*

We characterized heterogeneity in introgression across the genome among these same combinations and investigated whether differential introgression contributed variation potentially important in the divergence between species by calculating *f_d_* statistics in 50-kb sliding windows using a custom python script (modified from ABBABABA.py created by Simon H. Martin, available on https://github.com/simonhmartin/genomics_general; our modified version is provided in the supplementary materials of (Richards and Martin 2017)). We used the population allele frequencies of biallelic SNPs, allowing for a minimum of 100 variant sites with no missing data within a population per site. Linkage disequilibrium was calculated pairwise among SNPs across the largest linkage group in the genome (LG 3) using the “--r2” function in plink (Purcell et al. 2007). 50-kb is roughly the point at which linkage disequilibrium among all the groups in this dataset decays below a r^2^ value of 0.2 (Fig S12).

Significant regions of introgression were determined by calculating *f_d_* statistic across 150,000 windows containing 250 variants (average number of used variants in empirical windows) simulated under a coalescent model with no gene flow. Empirical windows were considered candidate introgressed regions if the *f_d_* statistic was above the maximum simulated *f_d_* value. Windows of variants were simulated for four populations using ms-move (Garrigan and Geneva 2014) and information about historical changes in population sizes were inferred from MSMC analyses (Schiffels and Durbin 2014). Simulations were run as follows: “msmove 8 1 -I 4 2 1 3 2 -g 1 40 -g 2 40 -g 3 1.59 -g 4 3.9 -eg 0.25 1 0 -eg 0.25 2 0 -eg 1.8 3 0 -eg 1 4 0 -en 2.5 1 0.1 -en 2.5 2 0.1 -en 6 3 0.5 -en 12.5 4 0.5 -T” for tests involving *S.galilaeus* MM and “msmove 8 1 -I 4 2 1 3 2 -g 1 40 -g 2 40 -g 3 1.59 -g 4 3.9 -eg 0.25 1 0 -eg 0.25 2 0 -eg 0.1 3 0 -eg 1 4 0 -en 2.5 1 0.1 -en 2.5 2 0.1 -en 0.4 3 0.167 -en 12.5 4 0.5 -T” for tests involving *S.galilaeus* CR . The simulations were then converted to sequence data using seq-gen and GTR model. These sequences were then inputted into the *fd* statistic pipeline

We ran MSMC on unphased genotypes from the 100 largest scaffolds (size range 0.86 Mb – 51.0 Mbp) for each individual separately. Genotypes were called from bam files using *samtools mpileup* with the following settings: minimum mapping quality of 20 (flag “-q 20”), minimum genotype quality of 20 (flag “-Q 20”) and a coefficient for downgrading mapping quality for reads containing excessive mismatches of 50 (flag “-C 50”). As recommended in the MSMC documentation (<https://github.com/stschiff/msmc-tools>), we used individual-specific masks to mask sites with less than half or more than double the mean coverage for that individual.  Nadachowska-brzyska et al. (2016) recommend to only use individuals with a mean coverage of at least 18, yet all our individuals were sequenced at a lower depth. After excluding individuals with depth below 7.5, (the *S. linelli* and *S. mongo* individuals), the genotypes used in this analysis had depths ranging from 7.52 to 16.67 (mean 9.97) across Barombi Mbo individuals, and genotypes for outgroup individuals had depths ranging from 8.10 to 16.97 (mean 11.75). While our MSMC results should therefore be interpreted with caution, the consistency among most individuals of the same species (Fig. S13-S14) suggests that the general patterns of the analysis are likely to be robust.

**Supplementary Tables**

**Table S1. Percentages of the genome assigned to topologies under various filtering criteria.** The relative percentage (out of all segments assigned in SAGUARO analysis) compared to the absolute percentage (out of the entire genome length 927,679,487) for each unique topology featuring either a monophyletic or non-monophyletic Barombi Mbo. We calculated both percentages under different filtering criteria of each segment assigned to a topology having at least one SNP, greater or equal to than 10 SNPs or 20 SNPs.

| **Monophyletic** | | | | | | |
| --- | --- | --- | --- | --- | --- | --- |
| Topology ID | **Relative Percentage** | | | **Absolute Percentage** | | |
|  | **None** | **10 SNPs** | **20 SNPs** | **None** | **10 SNPs** | **20 SNPs** |
| 1 | 0.172 | 0.049 | 0.023 | 0.151 | 0.033 | 0.012 |
| 2 | 0.045 | 0.042 | 0.017 | 0.039 | 0.028 | 0.009 |
| 4 | 0.246 | 0.189 | 0.168 | 0.216 | 0.128 | 0.090 |
| 6 | 1.078 | 0.279 | 0.135 | 0.948 | 0.189 | 0.072 |
| 11 | 0.344 | 0.093 | 0.044 | 0.303 | 0.063 | 0.024 |
| 12 | 0.374 | 0.125 | 0.091 | 0.329 | 0.085 | 0.049 |
| 15 | 0.726 | 0.246 | 0.170 | 0.639 | 0.167 | 0.091 |
| 16 | 0.994 | 0.319 | 0.210 | 0.875 | 0.216 | 0.113 |
| 19 | 0.407 | 0.153 | 0.100 | 0.358 | 0.104 | 0.054 |
| 20 | 1.677 | 1.581 | 1.572 | 1.476 | 1.072 | 0.844 |
| 26 | 0.703 | 0.371 | 0.224 | 0.619 | 0.252 | 0.120 |
| 29 | 0.172 | 0.096 | 0.067 | 0.152 | 0.065 | 0.036 |
| 35 | 0.855 | 0.247 | 0.147 | 0.752 | 0.167 | 0.079 |
| 42 | 0.061 | 0.035 | 0.027 | 0.054 | 0.024 | 0.014 |
| 46 | 0.249 | 0.283 | 0.269 | 0.219 | 0.192 | 0.145 |
| 54 | 3.504 | 1.735 | 1.003 | 3.084 | 1.176 | 0.538 |
| 55 | 0.070 | 0.033 | 0.024 | 0.061 | 0.023 | 0.013 |
| 60 | 0.060 | 0.044 | 0.037 | 0.053 | 0.030 | 0.020 |
| 62 | 0.051 | 0.028 | 0.017 | 0.045 | 0.019 | 0.009 |
| **Fig. 1B (65)** | **15.195** | **18.624** | **22.089** | **13.375** | **12.622** | **11.855** |
| 67 | 1.689 | 0.559 | 0.120 | 1.486 | 0.379 | 0.064 |
| 68 | 2.700 | 1.016 | 0.287 | 2.377 | 0.069 | 0.154 |
| **Fig. 1B (69)** | **63.184** | **71.224** | **71.340** | **55.614** | **48.271** | **38.289** |
| 70 | 0.045 | 0.024 | 0.019 | 0.039 | 0.016 | 0.010 |
| 71 | 1.508 | 0.762 | 0.613 | 1.327 | 0.517 | 0.329 |
| 72 | 0.036 | 0.017 | 0.010 | 0.032 | 0.012 | 0.006 |
| Total percentage | 94.6 | 98.17 | 98.23 | 84.62 | 65.92 | 53.04 |
| **Non-Monophyletic** | | | | | | |
| Topology ID | **Relative Percentage** | | | **Absolute Percentage** | | |
|  | **None** | **10 SNPs** | **20 SNPs** | **None** | **10 SNPs** | **20 SNPs** |
| 0 | 0.006 | 0.002 | 0.001 | 0.005 | 0.001 | 0.001 |
| **Fig. 2D (3)** | **0.224** | **0.087** | **0.061** | **0.197** | **0.059** | **0.033** |
| 5 | 0.058 | 0.053 | 0.027 | 0.051 | 0.036 | 0.014 |
| 7 | 0.147 | 0.042 | 0.024 | 0.130 | 0.029 | 0.013 |
| 8 | 0.057 | 0.024 | 0.021 | 0.050 | 0.016 | 0.011 |
| 9 | 0.029 | 0.022 | 0.022 | 0.025 | 0.015 | 0.012 |
| 10 | 0.121 | 0.048 | 0.029 | 0.107 | 0.033 | 0.015 |
| 13 | 0.145 | 0.065 | 0.040 | 0.127 | 0.044 | 0.021 |
| 14 | 0.141 | 0.070 | 0.055 | 0.124 | 0.047 | 0.029 |
| 17 | 0.305 | 0.152 | 0.107 | 0.269 | 0.103 | 0.058 |
| 18 | 0.095 | 0.042 | 0.031 | 0.084 | 0.028 | 0.016 |
| 21 | 0.096 | 0.038 | 0.021 | 0.085 | 0.026 | 0.011 |
| 22 | 0.044 | 0.030 | 0.024 | 0.039 | 0.020 | 0.013 |
| 23 | 0.076 | 0.037 | 0.018 | 0.067 | 0.025 | 0.010 |
| 24 | 0.043 | 0.017 | 0.010 | 0.038 | 0.011 | 0.005 |
| 25 | 0.128 | 0.047 | 0.026 | 0.113 | 0.032 | 0.014 |
| 27 | 0.059 | 0.031 | 0.022 | 0.052 | 0.021 | 0.012 |
| 28 | 0.057 | 0.035 | 0.025 | 0.050 | 0.024 | 0.013 |
| 30 | 0.096 | 0.048 | 0.036 | 0.084 | 0.032 | 0.019 |
| 31 | 0.038 | 0.017 | 0.009 | 0.033 | 0.012 | 0.005 |
| 32 | 0.051 | 0.040 | 0.016 | 0.045 | 0.027 | 0.008 |
| 33 | 0.065 | 0.026 | 0.016 | 0.058 | 0.018 | 0.008 |
| 34 | 0.045 | 0.032 | 0.021 | 0.040 | 0.022 | 0.011 |
| 36 | 0.042 | 0.030 | 0.026 | 0.037 | 0.021 | 0.014 |
| 37 | 0.053 | 0.023 | 0.017 | 0.047 | 0.016 | 0.009 |
| **Fig. 2C (38)** | **0.054** | **0.023** | **0.015** | **0.048** | **0.016** | **0.008** |
| **Fig. 2B (39)** | **0.101** | **0.041** | **0.026** | **0.089** | **0.028** | **0.014** |
| 40 | 0.048 | 0.025 | 0.019 | 0.042 | 0.017 | 0.010 |
| 41 | **0.110** | **0.052** | **0.035** | **0.097** | **0.035** | **0.019** |
| **Fig. 2A (43)** | **0.216** | **0.104** | **0.057** | **0.190** | **0.070** | **0.031** |
| 44 | 0.081 | 0.041 | 0.020 | 0.072 | 0.027 | 0.011 |
| 45 | 0.057 | 0.029 | 0.020 | 0.050 | 0.020 | 0.011 |
| 47 | 0.073 | 0.048 | 0.031 | 0.064 | 0.033 | 0.017 |
| 48 | 0.029 | 0.021 | 0.009 | 0.025 | 0.014 | 0.005 |
| 49 | 0.035 | 0.019 | 0.015 | 0.031 | 0.013 | 0.008 |
| 50 | 0.066 | 0.029 | 0.023 | 0.058 | 0.020 | 0.013 |
| 51 | 0.236 | 0.062 | 0.022 | 0.207 | 0.042 | 0.012 |
| 52 | 0.031 | 0.017 | 0.014 | 0.027 | 0.011 | 0.007 |
| 53 | 0.061 | 0.028 | 0.017 | 0.054 | 0.019 | 0.009 |
| 56 | 0.067 | 0.036 | 0.025 | 0.059 | 0.025 | 0.013 |
| 57 | 0.084 | 0.043 | 0.033 | 0.074 | 0.029 | 0.018 |
| 58 | 0.036 | 0.021 | 0.012 | 0.032 | 0.014 | 0.006 |
| 59 | 0.041 | 0.031 | 0.018 | 0.036 | 0.021 | 0.010 |
| 61 | 0.061 | 0.023 | 0.015 | 0.054 | 0.015 | 0.008 |
| 63 | 0.048 | 0.023 | 0.016 | 0.043 | 0.016 | 0.009 |
| 64 | 0.034 | 0.018 | 0.011 | 0.030 | 0.012 | 0.006 |
| 66 | 0.043 | 0.020 | 0.014 | 0.038 | 0.014 | 0.008 |
| 73 | 0.014 | 0.006 | 0.003 | 0.012 | 0.004 | 0.002 |
| 74 | 0.009 | 0.006 | 0.004 | 0.008 | 0.004 | 0.002 |
| Total Percentage | 3.85 | 1.82 | 1.18 | 3.39 | 1.24 | 0.632 |

**Table S2. Overlap of *Saguaro* and *f_d_* introgressed regions.** The cumulative length of regions assigned by *Saguaro* to the supported polyphyletic topologies (Fig 2) and the percentage of the significant *f_d_* regions that were assigned to these topologies.

| Linkage Group | *f_d_* region position | Cumulative length of *Saguaro* regions | Percentage |
| --- | --- | --- | --- |
| Regions uniquely introgressed into a single species | | | |
| LG 3 | 14525001-14575000 | 165 | 0.3300066 |
| LG 4 | 800001-855000 | 697 | 1.2672958 |
| LG 5 | 35530001-35595000 | 103 | 0.158464 |
| LG 6 | 10670001-10740000 | 554 | 0.7914399 |
| LG 6 | 20935001-10740000 | 5351 | 5.9456216 |
| LG 6 | 22460001-22515000 | 1553 | 2.8236877 |
| LG 11 | 28280001-28375000 | 115 | 0.1210539 |
| LG 16-21 | 31140001-31220000 | 2197 | 2.7462843 |
| LG 17 | 9260001-9315000 | 76 | 0.1381843 |
| LG 20 | 150001-230000 | 1389 | 1.7362717 |
| LG 20 | 1705001-1755000 | 6662 | 13.3242665 |
| LG 20 | 19570001-19630000 | 483 | 0.8050134 |
| NT_167475.1 | 450001-500000 | 5530 | 11.0602212 |
| NT_167500.1 | 930001-1000000 | 7074 | 10.1058587 |
| NT_167557.1 | 1410001-1495000 | 8877 | 10.4436523 |
| NT_167586.1 | 220001-290000 | 8286 | 11.837312 |
| NT_167617.1 | 320001-400000 | 34957 | 43.6967962 |
| NT_167636.1 | 140001-190000 | 339 | 0.6780136 |
| NT_167663.1 | 535001-630000 | 4933 | 5.1926862 |
| NT_167671.1 | 265001-330000 | 4257 | 6.5493315 |
| NT_167675.1 | 605001-655000 | 189 | 0.3780076 |
| NT_167747.1 | 45001-330000 | 8788 | 3.0835196 |
| NT_168010.1 | 30001-80000 | 275 | 0.550011 |
| NT_168013.1 | 1-70000 | 3721 | 5.3157902 |
| Regions of shared introgression within a subclade | | | |
| LG 2 | 9190001-9275000 | 3730 | 4.3882869 |
| NT_167557.1 | 1110001-1170000 | 8085 | 13.4752246 |
| NT_167617.1 | 105001-160000 | 2256 | 4.1018928 |
| NT_167653.1 | 680001-770000 | 18279 | 20.3102257 |
| NT_167679.1 | 395001-445000 | 1249 | 2.49805 |
| NT_167702.1 | 445001-530000 | 363 | 0.4270638 |
| NT_167728.1 | 40001-120000 | 73 | 0.0912511 |
| NT_167736.1 | 160001-220000 | 484 | 0.8066801 |
| Regions of shared introgression within Barombi Mbo | | | |
| LG 3 | 1185001-1235000 | 138 | 0.2760055 |
| LG 3 | 3325001-3425000 | 19766 | 19.7661977 |
| LG 5 | 35510001-35575000 | 334 | 0.5138541 |
| LG 17 | 24560001-24620000 | 812 | 1.3533559 |
| LG 20 | 19550001-19600000 | 2716 | 5.4321086 |
| LG 20 | 300001-370000 | 2455 | 3.507193 |
| NT_167475.1 | 2260001-2340000 | 10186 | 12.7326592 |
| NT_167535.1 | 855001-925000 | 9610 | 13.7287676 |
| NT_167709.1 | 135001-190000 | 1672 | 3.0400553 |
| NT_167716.1 | 100001-265000 | 10662 | 6.4618573 |
| NT_167744.1 | 260001-340000 | 8070 | 10.0876261 |
| NT_167891.1 | 55001-125000 | 454 | 0.6485807 |
| NT_167891.1 | 75001-130000 | 1549 | 2.8164148 |

**Table S3. Average *D_xy_* among Barombi Mbo and riverine *Sarotherodon* species across the genome.**

|  | *K. eisentrauti* | *K. dikume* | *P. maclareni* | *S. mongo* | *S. mariae* | *S. pindu* | *M.*  *myaka* | *S. linnelli* | *S. galilaeus* MM | *S. galilaeus* CR |
| --- | --- | --- | --- | --- | --- | --- | --- | --- | --- | --- |
| *K. eisentrauti* | -- | 0.00062 | 0.00079 | 0.00083 | 0.00087 | 0.00081 | 0.00089 | 0.00081 | 0.00801 | 0.0015 |
| *K. dikume* |  | -- | 0.00086 | 0.00088 | 0.00092 | 0.00087 | 0.00094 | 0.00087 | 0.00794 | 0.00154 |
| *P. maclareni* |  |  | -- | 0.00088 | 0.00092 | 0.00086 | 0.00094 | 0.00082 | 0.00788 | 0.00152 |
| *S. mongo* |  |  |  | -- | 0.00071 | 0.00059 | 0.00092 | 0.00088 | 0.00782 | 0.00152 |
| *S. mariae* |  |  |  |  | -- | 0.00068 | 0.00097 | 0.00091 | 0.00789 | 0.00156 |
| *S. pindu* |  |  |  |  |  | -- | 0.00091 | 0.00085 | 0.008 | 0.00152 |
| *M.myaka* |  |  |  |  |  |  | -- | 0.00091 | 0.0078 | 0.00152 |
| *S. linnelli* |  |  |  |  |  |  |  | -- | 0.0077 | 0.00149 |
| *S. galilaeus* MM |  |  |  |  |  |  |  |  | -- | 0.0076 |
| *S. galilaeus* CR |  |  |  |  |  |  |  |  |  | -- |

**Table S4. Candidate introgressed regions in Barombi Mbo cichlid radiation that are shared across multiple species in a subclade**. These regions feature significant *f_d_* values between riverine populations of *S. galilaeus* MM (Mungo and Meme River) and the three subclades of the radiation focused on in this study. Unannotated regions with no GO terms are marked with (-).

| Linkage Group | Position | Gene(s) | Gene Ontology Terms |
| --- | --- | --- | --- |
| Konia | | | |
| NC_022200.1 | 9190001-9275000 | UPC(6) | -- |
| NT_167557.1 | 1110001-1170000 | fam111a; rnf175; trim2 | fibrillar center, chromatin;ubiquitin protein ligase activity; ubiquitin-protein transferase activity |
| NT_167679.1 | 395001-445000 | -- | -- |
| NT_167702.1 | 445001-530000 | lipe; cd79a; arhgef1 | triglyceride catabolic process;B cell receptor signaling pathway;regulation of Rho protein signal transduction |
| NT_167891.1 | 155001-225000 | cxcr3; pafah1b3*; cnfn;  tlr13 ; hmcn1; ceacam20;  ceacam5; UPC | CXCR3 chemokine receptor binding;platelet-activating factor acetyltransferase activity, spermatogenesis;cornified envelope,keratinization;toll-like receptor 13 signaling pathway;basement membrane; fin morphogenesis;positive regulation of cytokine production;negative regulation of myotube differentiation |
| NC_022205.1 | 1390001-1445000 | -- | -- |
| NT_167617.1 | 105001-160000 | gastrula zinc finger XICGF26.1 | unknown |
| NT_167653.1 | 680001-770000 | dock3 | small GTPase mediated signal transduction |
| NT_167736.1 | 160001-220000 | btnl2; UPC(3) | negative regulation of T cell receptor signaling pathway |
| Stomatepia | | | |
| NT_167728.1 | 40001-120000 | H2-Q10; H2-LHX9; tnc | ribonuclease H2 complex;ribonuclease H2 complex;prostate gland epithelium morphogenesis |

*best candidate region for secondary gene flow contributing to diversification; UPC = uncharacterized protein coding gene

**Table S5. Candidate introgressed regions in Barombi Mbo cichlid radiation that are shared across multiple species across Barombi Mbo**. These regions feature significant *f_d_* values between riverine populations of *S. galilaeus* MM (Mungo and Meme River) and the three subclades of the radiation focused on in this study. Unannotated regions with no GO terms are marked with (-).

| Linkage Group | Position | Gene(s) | Gene Ontology Terms |
| --- | --- | --- | --- |
| LG1 | 26250001-26315000 | adgrg1 | heparin binding |
| LG3 | 3325001-3425000 | UPC(9); UPG (1) | -- |
| LG3 | 1185001-1240000 | sh3tc1 | biological process |
| LG5 | 35500001-35575000 | or52e8*;matn4 | olfactory receptor activity;growth plate cartilage chondrocyte morphogenesis,calcium ion binding |
| LG16-21 | 5365001-5420000 | -- | -- |
| LG17 | 24560001-24620000 | prdm4;tmem209;ahcyl2 | histone methyltransferase binding*;integral component of membrane;S-adenosylmethionine cycle activity |
| LG18 | 20715001-20770000 | tep1;znrf1;grina;parp2;UPC | RNA binding; E3 ubiquitin protein ligase activity;negative regulation of endoplasmic reticulum stress-induced intrinsic apoptotic signaling pathway;DNA ligation involved in DNA repair |
| LG20 | 19300001-19410000 | klhdc8b | ubiquitin-protein transferase activity |
| LG20 | 19550001-19615000 | sbk; UPC | protein serine/threonine kinase activity |
| NT_167475.1 | 2260001-2340000 | serp1 ;C-type natriuretic peptide 2;eno3;pfn1*;UPC(2) | endoplasmic reticulum unfolded protein response ;unknown;phosphopyruvate hydratase activity;positive regulation of actin filament bundle assembly*;-- |
| NT_167535.1 | 855001-930000 | dmbt1 | defense response to Gram-negative bacterium (Digestive Phnotype |
| NT_167709.1 | 135001-230000 | hepacam | cell-cell junction; cell adhesion |
| NT_167716.1 | 95001-260000 | st8sia5 | sialylation,sialyltransferase activity |
| NT_167744.1 | 260001-340000 | siglec1;gpr87;p2ry14 | regulation of osteoclast development;G-protein coupled receptor signaling pathway*;G-protein coupled receptor signaling pathway |
| NT_167891.1 | 55001-130000 | -- | -- |
| NT_167927.1 | 1-55000 | mdn1* | rRNA processing |

*best candidate region for secondary gene flow contributing to diversification; UPC = uncharacterized protein coding gene

**Table S6. Within-population genetic diversity in introgressed regions in Barombi Mbo and *Sarotherodon* riverine populations**. Bolded rows indicate introgressed regions where with-in population nucleotide diversity is >10X lower than the scaffold average.

| ***Konia + Pungu*** | | | | | | | | | | | |
| --- | --- | --- | --- | --- | --- | --- | --- | --- | --- | --- | --- |
|  |  | ***K.eisentrauti*** | | ***K.dikume*** | | ***P. maclareni*** | | ***S. galilaeus* MM** | | ***S. galilaeus* CR** | |
| **scaffold** | **position** | ***f_d_* region** | **LG avg** | ***f_d_* region** | **LG avg** | ***f_d_* region** | **LG avg** | ***f_d_* region** | **LG avg** | ***f_d_* region** | **LG avg** |
| *K. dikume* introgressed regions | | | | | | | | | | | |
| NC_022200.1 | 18660001-18710000 | 0.00054383 | 0.0004 | 0.00178761 | 0.0004 | 0.00042413 | 0.0004 | 0.00314588 | 0.0004 | 0.00349716 | 0.001 |
| NT_168013.1 | 1-70000 | 0.00068225 | 0.0007 | 0.00068225 | 0.001 | 0.00068225 | 0.0007 | 0.00068225 | 0.002 | 0.00068225 | 0.001 |
| ***K. eisentrauti* introgressed regions** | | | | | | | | | | | |
| NC_022204.1 | 22460001-22515000 | 0.00145323 | 0.0004 | 0.00115539 | 0.0004 | 0.00174443 | 0.0004 | 0.00116517 | 0.0005 | 0.00096542 | 0.001 |
| NT_167586.1 | 220001-  290000 | 0.0008931 | 0.001 | 0.00120841 | 0.001 | 0.00095027 | 0.001 | 0.00166443 | 0.002 | 0.00171494 | 0.002 |
| NT_167663.1 | 535001-  630000 | 0.00059447 | 0.0005 | 0.00056152 | 0.0005 | 0.00062971 | 0.0005 | 0.00034735 | 0.00032 | 0.00565028 | 0.0003 |
| ***P. maclareni* introgressed regions** | | | | | | | | | | | |
| NC_022201.1 | 14525001-14575000 | 0.0026372 | 0.0008 | 0.00230363 | 0.0008 | 0.00218461 | 0.0008 | 0.00218618 | 0.0008 | 0.00302087 | 0.001 |
| NC_022215.1 | 9260001-9315000 | 0.00367677 | 0.0004 | 0.00308452 | 0.0004 | 0.00194999 | 0.0004 | 0.00049984 | 0.0004 | 0.00263629 | 0.0004 |
| NT_167557.1 | 1410001-1495000 | 0.00185095 | 0.002 | 0.0016627 | 0.001 | 0.0014918 | 0.001 | 0.0011726 | 0.001 | 0.00279756 | 0.002 |
| NT_167671.1 | 265001-330000 | 0.00272164 | 0.001 | 0.00235028 | 0.001 | 0.00187547 | 0.001 | 0.00128621 | 0.002 | 0.00281824 | 0.002 |
| **NT_168010.1** | **30001-80000** | **6.86E-05** | **0.0001** | **0.00052284** | **0.001** | **0.00080094** | **0.001** | **0.00060812** | **0.0006** | **0.0016056** | **0.001** |
| ***Stomatepia*** | | | | | | | | | | | |
|  |  | ***S. mariae*** | | ***S. pindu*** | | ***S. mongo*** | | ***S. galilaeus* MM** | | ***S. galilaeus* CR** | |
| **scaffold** | **position** | ***f_d_* region** | **LG avg** | ***f_d_* region** | **LG avg** | ***f_d_* region** | **LG avg** | ***f_d_* region** | **LG avg** | ***f_d_* region** | **LG avg** |
| ***S. mongo* introgressed regions** | | | | | | | | | | | |
| **NC_022218.1** | **150001-230000** | **8.5296E-05** | **0.004** | **0.00143164** | **0.004** | **0** | **0.004** | **0.0007122** | **0.004** | **0.00205955** | **0.001** |
| ***S. mariae* introgressed region** | | | | | | | | | | | |
| NC_022214.1 | 31140001-31220000 | 0.00118162 | 0.004 | 0.00149186 | 0.004 | 0.00104205 | 0.004 | 0.00094478 | 0.004 | 0.00232024 | 0.001 |
| ***S. pindu* introgressed region** | | | | | | | | | | | |
| **NT_167675.1** | **605001-655000** | **0.00272135** | **0.0008** | **0.00056357** | **0.005** | **0.003945** | **0.001** | **0.00109433** | **0.0008** | **0.00369409** | **0.001** |
| ***Myaka* + *Sarotherodon*** | | | | | | | | | | | |
|  |  | ***M. myaka*** | | ***S. linnelli*** | | ***S. galilaeus* MM** | | ***S. galilaeus* CR** | |  |  |
| **scaffold** | **position** | ***f_d_* region** | **LG avg** | ***f_d_* region** | **LG avg** | ***f_d_* region** | **LG avg** | ***f_d_* region** | **LG avg** |  |  |
| NC_022201.1 | 17560001-17610000 | 0.00201684 | 0.001 | 0.00198132 | 0.001 | 0.0010686 | 0.0007 | 0.00362337 | 0.001 |  |  |
| NC_022202.1 | 800001-855000 | 0.00747169 | 0.0006 | 0.0092404 | 0.0005 | 0.00657196 | 0.0005 | 0.00825817 | 0.001 |  |  |
| NC_022203.1 | 35530001-35595000 | 0.00490386 | 0.0005 | 0.013727 | 0.0005 | 0.00974243 | 0.0004 | 0.00778364 | 0.001 |  |  |
| NC_022204.1 | 10670001-10740000 | 0.00747169 | 0.0006 | 0.0092404 | 0.0005 | 0.00657196 | 0.0005 | 0.00825817 | 0.001 |  |  |
| NC_022204.1 | 20935001-21025000 | 0.00253494 | 0.0006 | 0.0026205 | 0.0005 | 0.00288848 | 0.0005 | 0.00306214 | 0.001 |  |  |
| NC_022209.1 | 28280001-28375000 | 0.00356265 | 0.0005 | 0.00229944 | 0.0005 | 0.00138885 | 0.0005 | 0.0049961 | 0.001 |  |  |
| NC_022218.1 | 1705001-1755000 | 0.00095566 | 0.0006 | 0.00149491 | 0.0005 | 0.00099938 | 0.0003 | 0.00207371 | 0.001 |  |  |
| NC_022218.1 | 19570001-19630000 | 0.0006839 | 0.0006 | 0.00099304 | 0.0005 | 0.00015891 | 0.0003 | 0.00155764 | 0.001 |  |  |
| NT_167475.1 | 450001-500000 | 0.00356569 | 0.0024 | 0.00619587 | 0.0029 | 0.00512233 | 0.0024 | 0.00201672 | 0.003 |  |  |
| NT_167500.1 | 835001-1000000 | 0.0028197 | 0.0009 | 0.00074963 | 0.0006 | 0.00111938 | 0.0006 | 0.00401496 | 0.002 |  |  |
| NT_167568.1 | 10001-60000 | 0.00296742 | 0.002 | 0.0031527 | 0.001 | 0.00238853 | 0.002 | 0.00266981 | 0.001 |  |  |
| NT_167617.1 | 320001-400000 | 0.00111105 | 0.002 | 0.00013603 | 0.002 | 0.00046038 | 0.002 | 0.00137309 | 0.002 |  |  |
| NT_167716.1 | 220001-270000 | 0.00505633 | 0.002 | 0.01043704 | 0.004 | 0.00528548 | 0.004 | 0.00753241 | 0.002 |  |  |
| NT_167790.1 | 255001-305000 | 0.00458413 | 0.001 | 0.0037551 | 0.001 | 0.00379285 |  | 0.00400954 | 0.001 |  |  |
| NT_167636.1 | 140001-190000 | 0.0059642 | 0.003 | 0.00871405 | 0.003 | 0.00564376 | 0.003 | 0.00417817 | 0.003 |  |  |

**References**

Danecek, P., A. Auton, G. Abecasis, C. A. Albers, E. Banks, M. A. DePristo, R. E. Handsaker, G. Lunter, G. T. Marth, S. T. Sherry, G. McVean, and R. Durbin. 2011. The variant call format and VCFtools. Bioinformatics 27:2156–2158.

DePristo, M. A., E. Banks, R. Poplin, K. V Garimella, J. R. Maguire, C. Hartl, A. A. Philippakis, G. del Angel, M. A. Rivas, M. Hanna, A. McKenna, T. J. Fennell, A. M. Kernytsky, A. Y. Sivachenko, K. Cibulskis, S. B. Gabriel, D. Altshuler, and M. J. Daly. 2011. A framework for variation discovery and genotyping using next-generation DNA sequencing data. Nat. Genet. 43:491–8.

Garrigan, D., and A. Geneva. 2014. msmove: A modified version of Hudson’s coalescent simulator ms allowing for finer control and tracking of migrant genealogies.

Guo, B., F. J. J. Chain, E. Bornberg-Bauer, E. H. Leder, and J. Merilä. 2013. Genomic divergence between nine- and three-spined sticklebacks. BMC Genomics 14:756.

Li, H. 2013. Aligning sequence reads, clone sequences and assembly contigs with BWA-MEM. arXiv 1303:1–3.

Li, H., and R. Durbin. 2009. Fast and accurate short read alignment with Burrows-Wheeler transform. Bioinformatics 25:1754–1760.

Marsden, C. D., Y. Lee, K. Kreppel, A. Weakley, A. Cornel, H. M. Ferguson, E. Eskin, and G. C. Lanzaro. 2014. Diversity, differentiation, and linkage disequilibrium: prospects for association mapping in the malaria vector Anopheles arabiensis. G3 (Bethesda). 4:121–31.

Purcell, S., B. Neale, K. Todd-Brown, L. Thomas, M. A. R. Ferreira, D. Bender, J. Maller, P. Sklar, P. I. W. de Bakker, M. J. Daly, and P. C. Sham. 2007. PLINK: A Tool Set for Whole-Genome Association and Population-Based Linkage Analyses. Am. J. Hum. Genet. 81:559–575.

Recknagel, H., K. R. Elmer, and A. Meyer. 2013. A hybrid genetic linkage map of two ecologically and morphologically divergent Midas cichlid fishes (Amphilophus spp.) obtained by massively parallel DNA sequencing (ddRADSeq). G3 (Bethesda). 3:65–74.

Richards, E. J., and C. H. Martin. 2017. Adaptive introgression from distant Caribbean islands contributed to the diversification of a microendemic adaptive radiation of trophic specialist pupfishes. PLOS Genet. 13:e1006919. Public Library of Science.

Schiffels, S., and R. Durbin. 2014. Inferring human population size and separation history from multiple genome sequences. Nat. Genet. 46:919. Nature Publishing Group, a division of Macmillan Publishers Limited. All Rights Reserved.

Schliewen, U. K., D. Tautz, and S. Paabo. 1994. Sympatric speciation suggested by monophyly of crater lake cichlids. Nature 368:629–631.

Van der Auwera, G. A., M. 0. Carneiro, C. Hartl, R. Poplin, G. del Angel, A. Levy‐Moonshine, T. Jordan, K. Shakir, D. Roazen, J. Thibault, E. Banks, G. V. Kiran, D. Altshuler, S. Gabriel, and M. A. DePristo. 2013. From FastQ Data to High‐Confidence Variant Calls: The Genome Analysis Toolkit Best Practices Pipeline. Curr. Protoc. Bioinforma. 43:11.10.1-11.10.33. Wiley-Blackwell.

Zamani, N., P. Russell, H. Lantz, M. P. Hoeppner, J. R. Meadows, N. Vijay, E. Mauceli, F. Di Palma, K. Lindblad-Toh, P. Jern, and M. G. Grabherr. 2013. Unsupervised genome-wide recognition of local relationship patterns. BMC Genomics 14:347.
