# Supplementary figures and images for: Don't throw out the sympatric speciation with the crater lake water: fine‐scale investigation of introgression provides equivocal support for causal role of secondary gene flow in one of the clearest examples of sympatric speciation
Source: Evol Lett. 2018 Aug 15;2(5):524–40. doi: 10.1002/evl3.78 (PMC6145409; doi:10.1002/evl3.78)

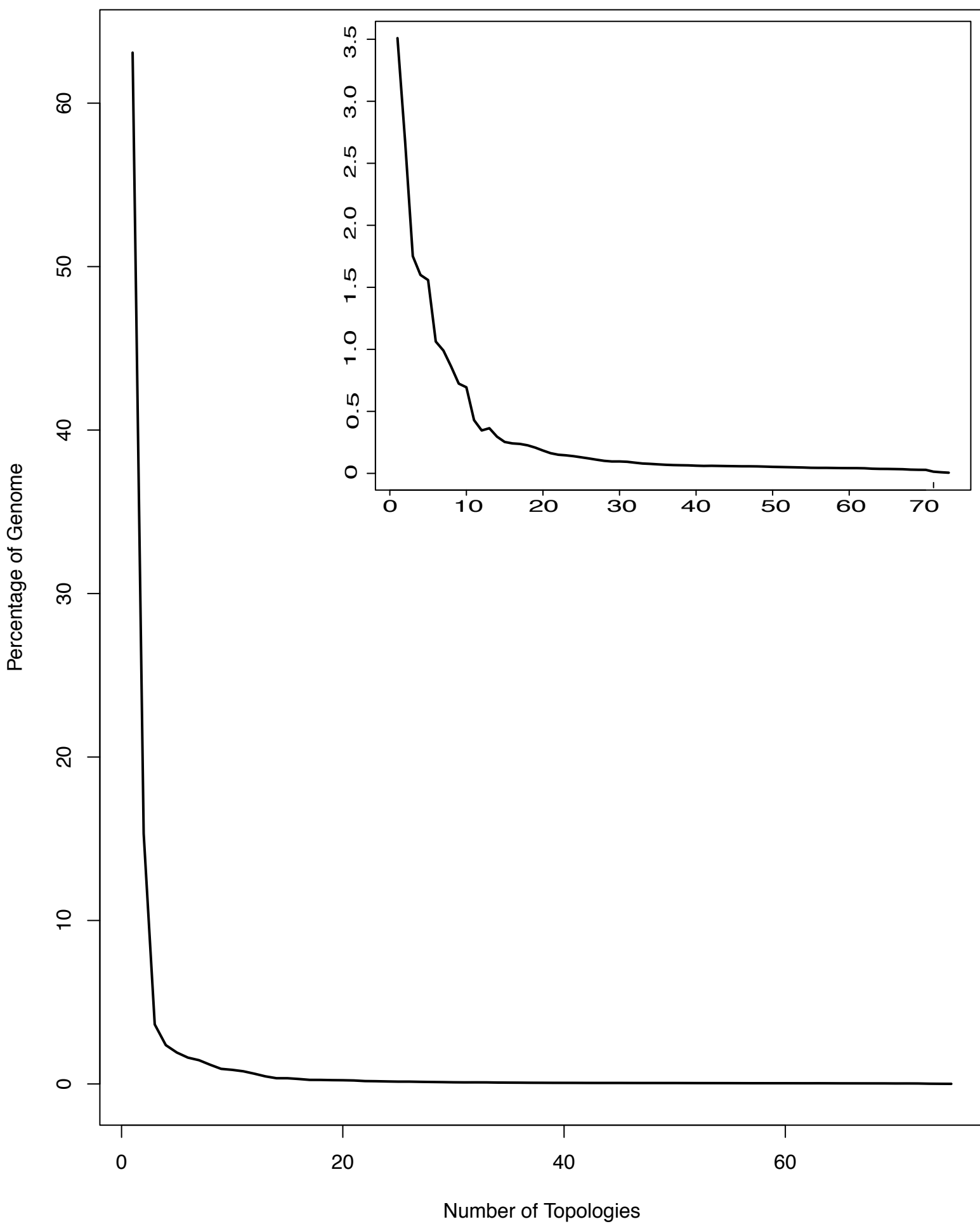

Supplement: Supplementary file 2 — Figure S1. The percentage of the genome assigned to each topology by Saguaro. [file EVL3-2-524-s002.pdf]

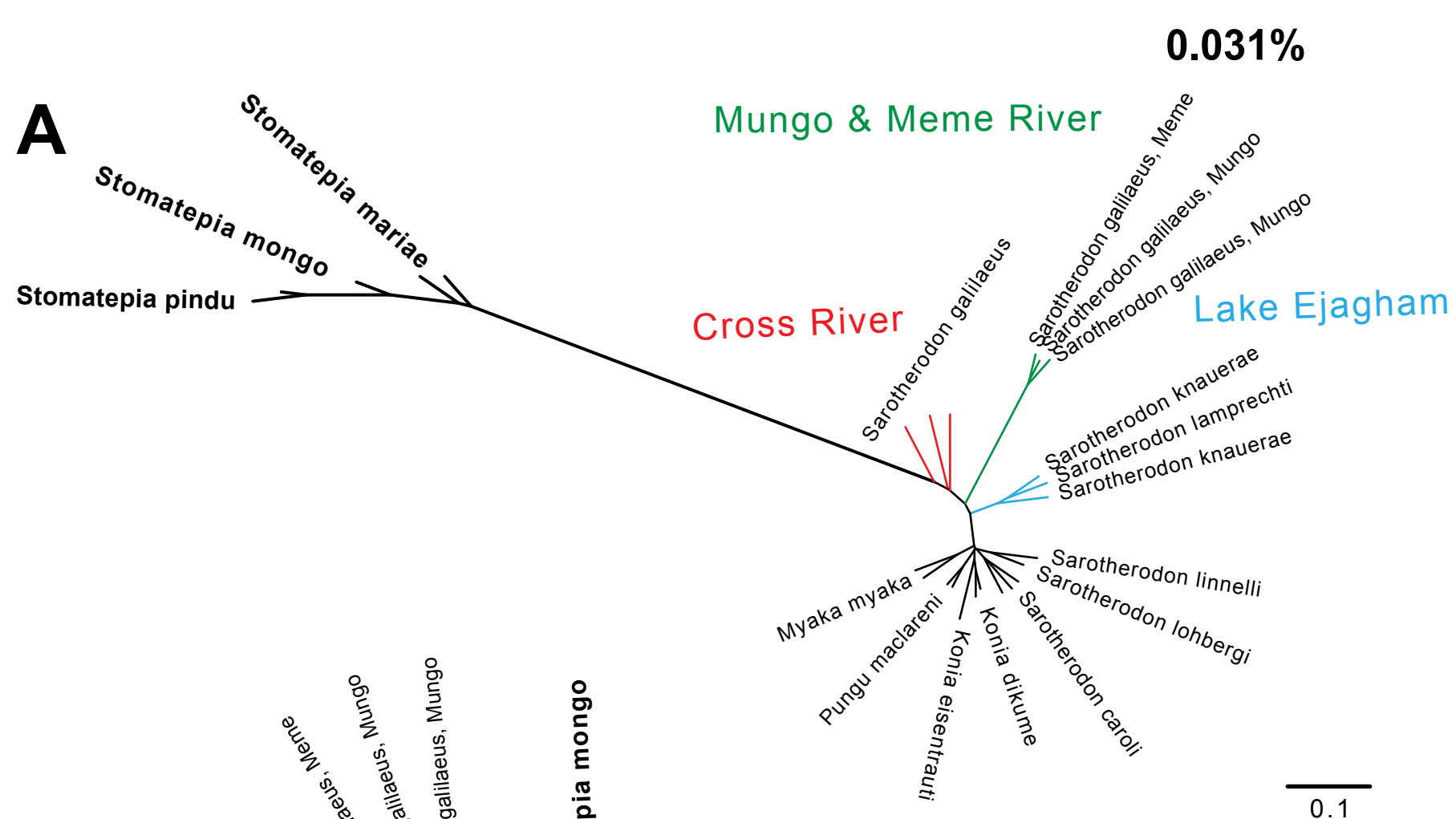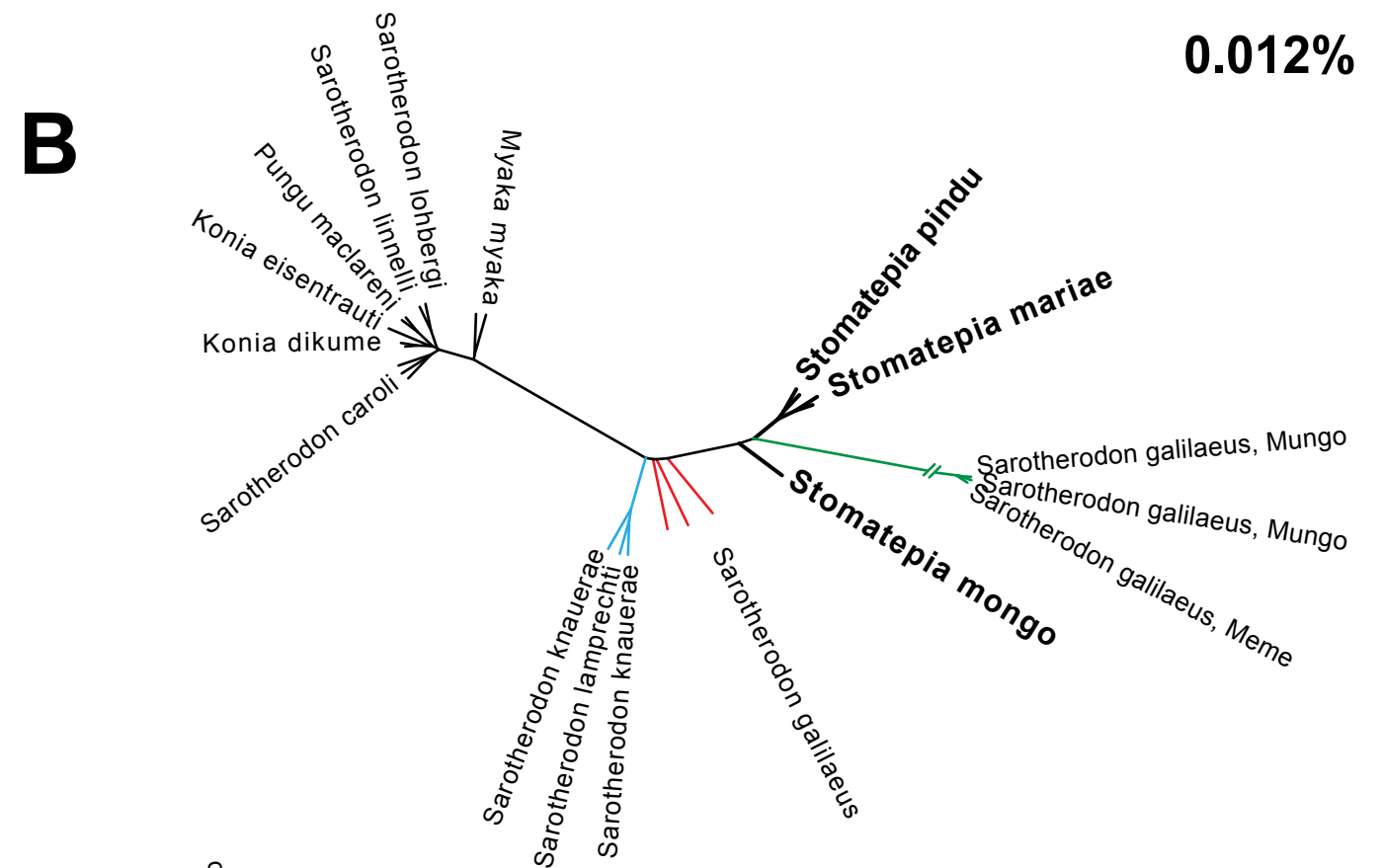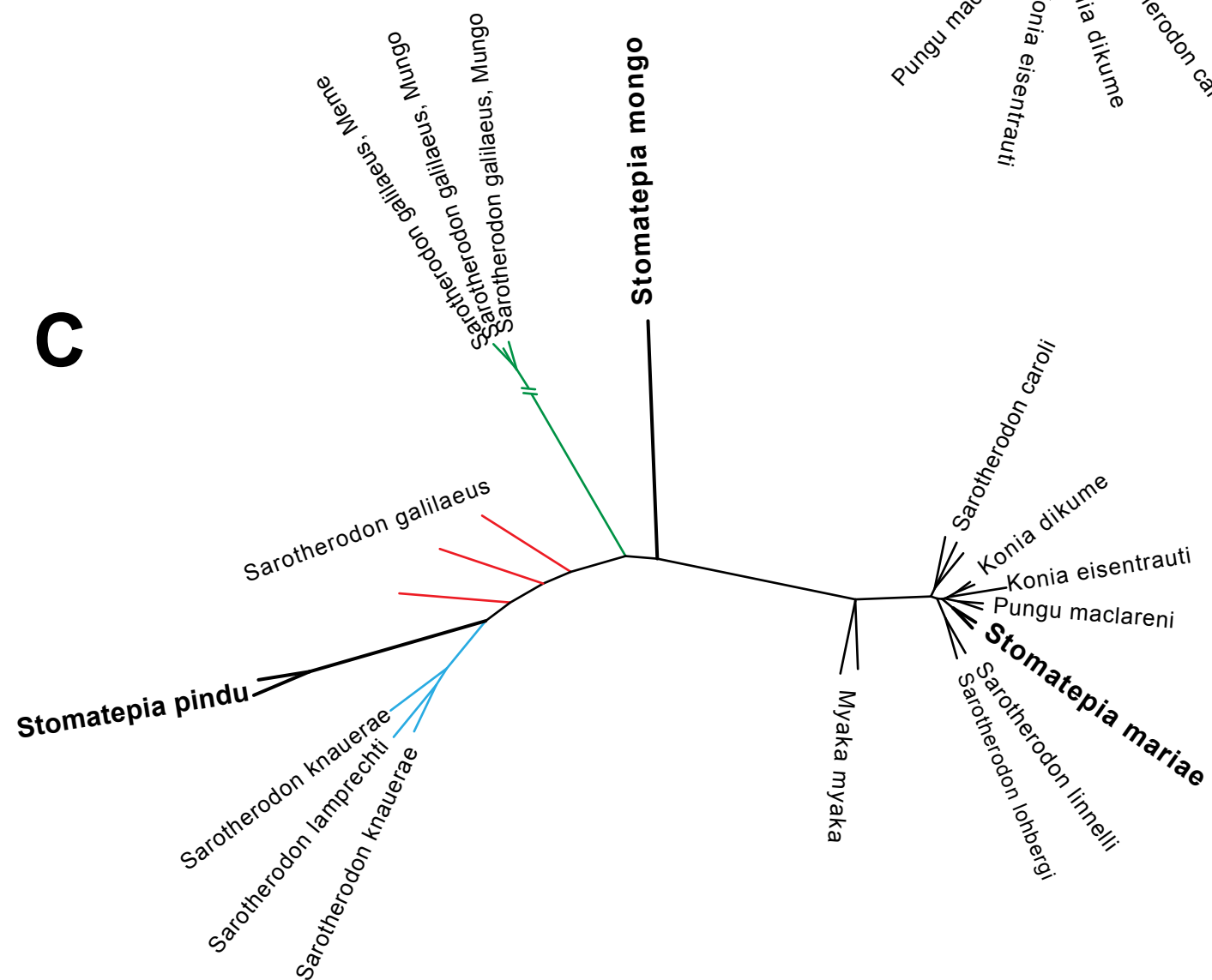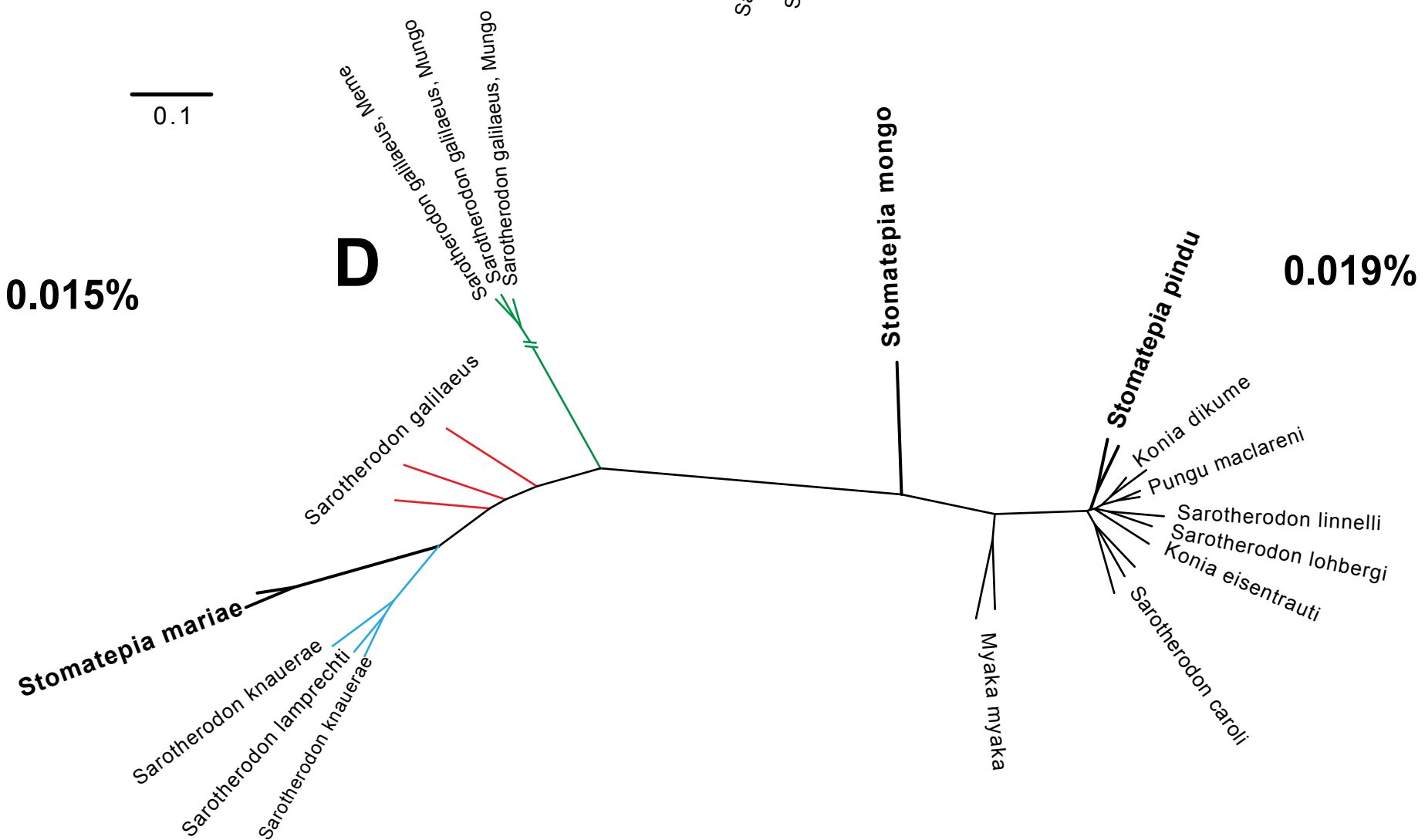

Supplement: Supplementary file 3 — Figure S2. Saguaro topologies featuring Barombi Mbo polyphyly with riverine populations involving the Stomatepia three‐species complex. [file EVL3-2-524-s003.pdf]

**A**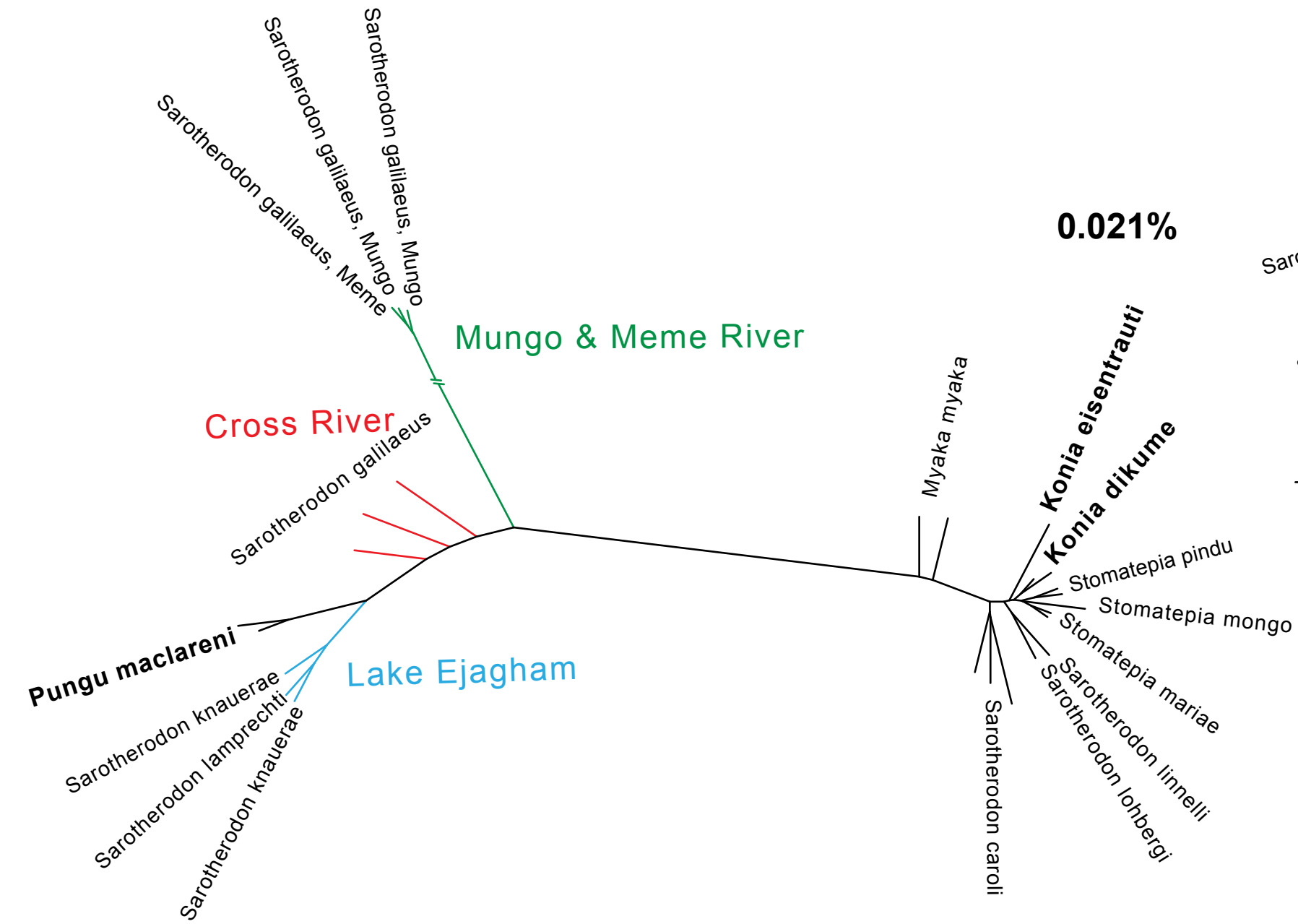**B**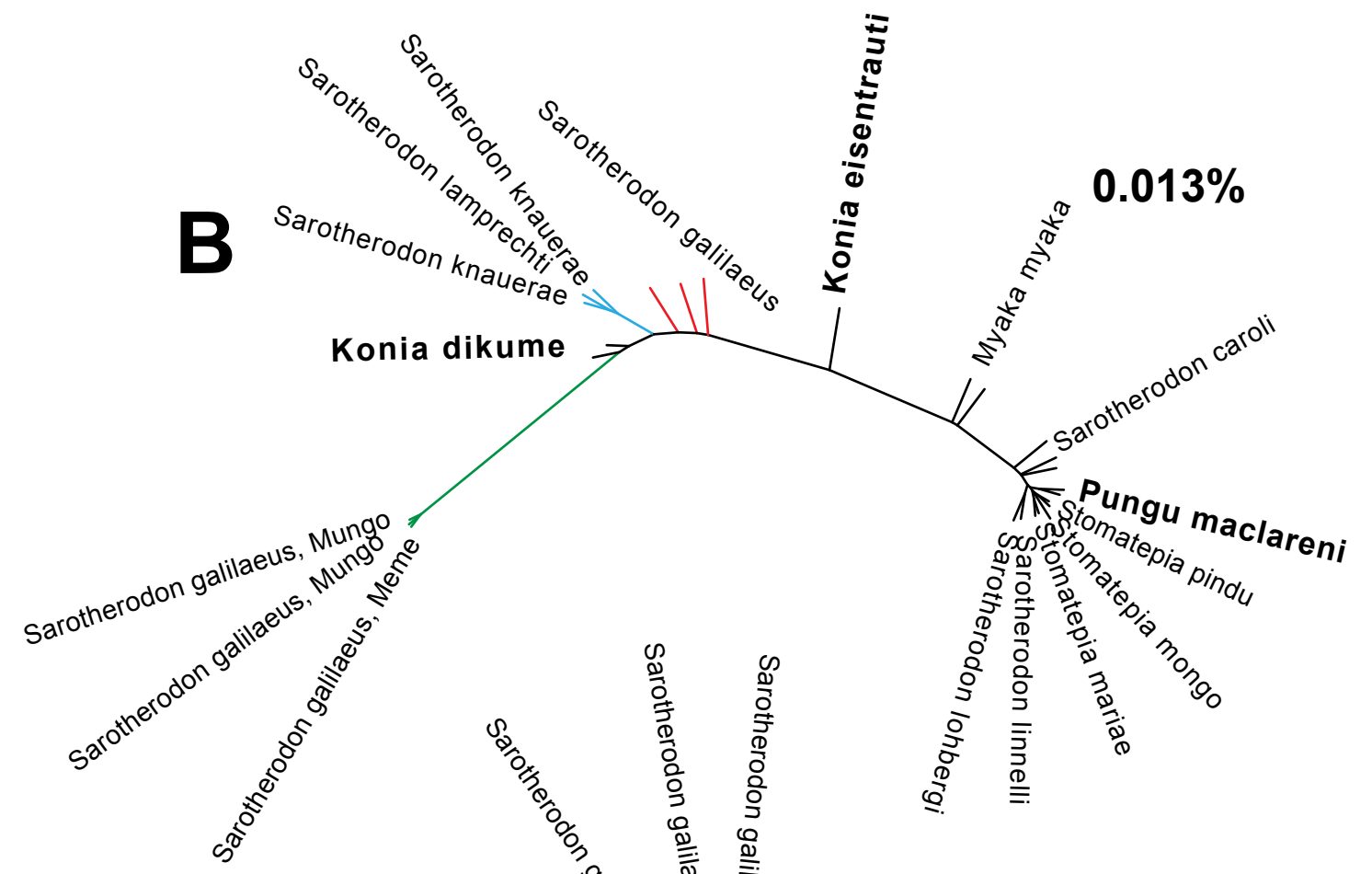**C**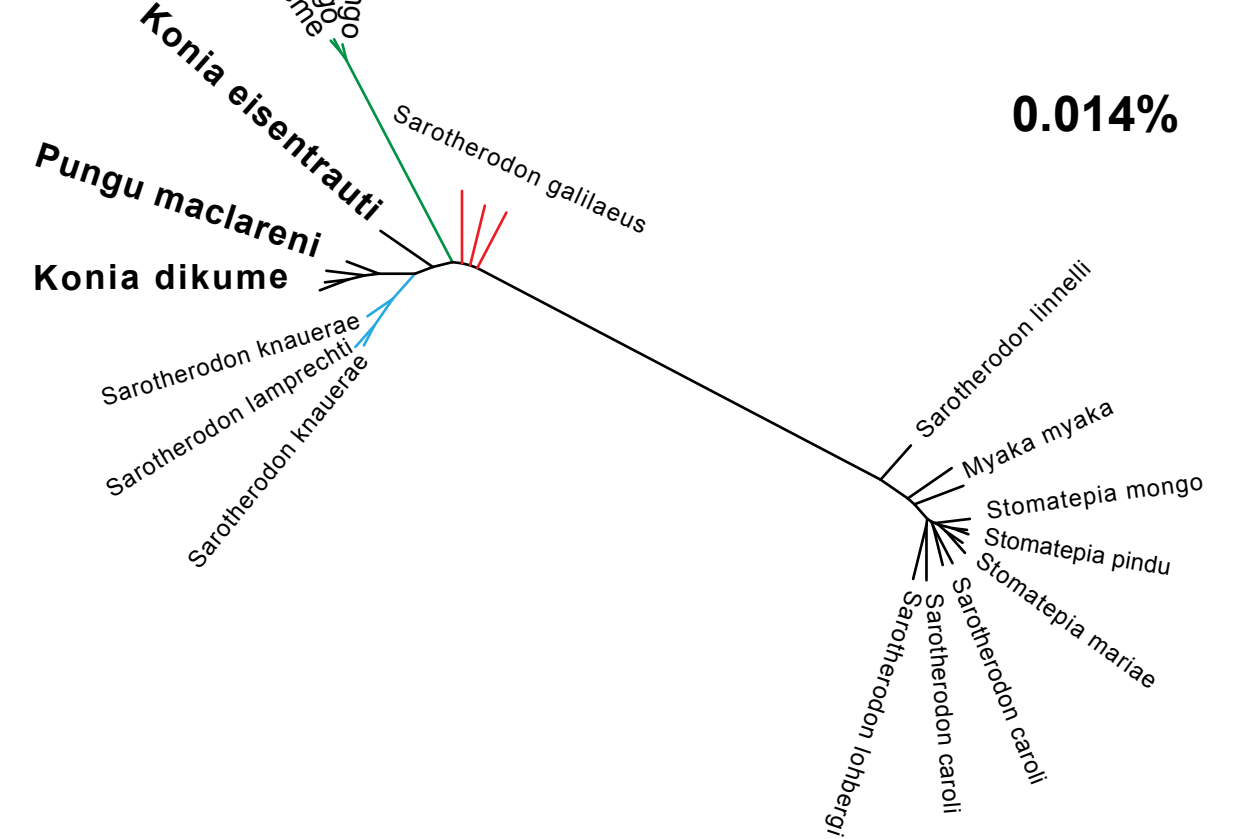

Supplement: Supplementary file 4 — Figure S3. Saguaro topologies featuring Barombi Mbo polyphyly with riverine populations involving the Konia + Pungu subclade. [file EVL3-2-524-s004.pdf]

**A**

Mungo &amp; Meme River

Cross River

0.058%

**B**

Lake Ejagham

0.033%

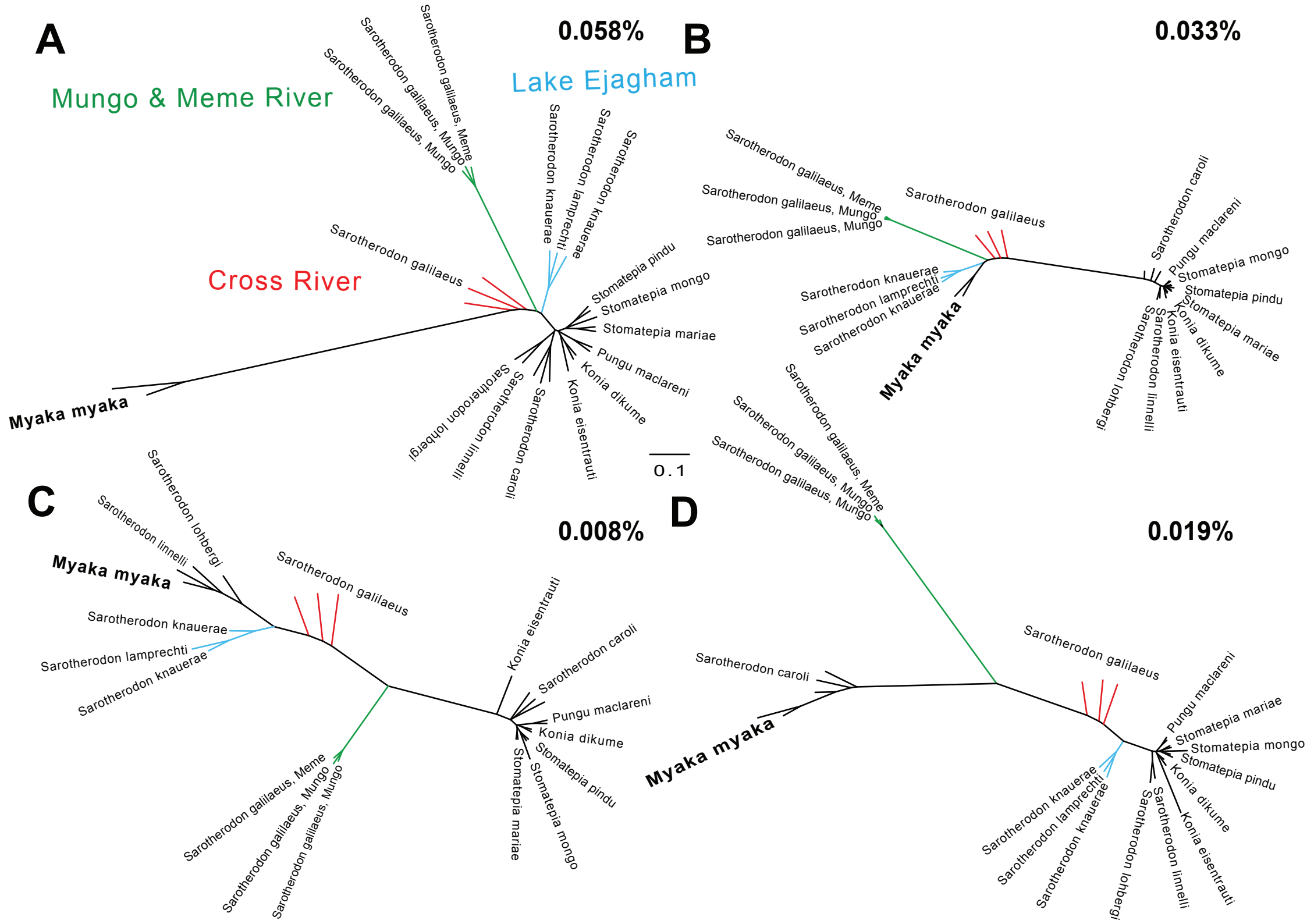**C****Myaka myaka**

0.008%

**D**

0.019%

**Myaka myaka**

Supplement: Supplementary file 5 — Figure S4. Saguaro topologies featuring Barombi Mbo polyphyly with riverine populations involving the Myaka + Sarotherodon subclade. [file EVL3-2-524-s005.pdf]

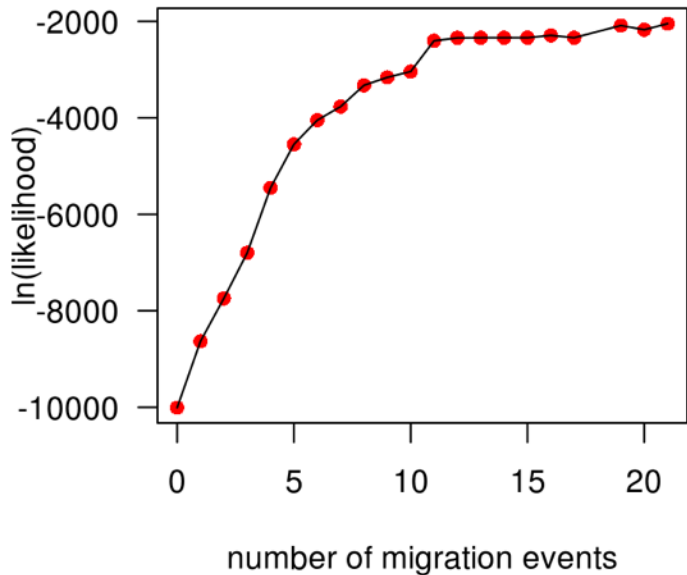

Supplement: Supplementary file 6 — Figure S5. The log‐likelihood of TreeMix population graphs for Barombi Mbo cichlids as a function of the number of migration events. [file EVL3-2-524-s006.pdf]

A

*S. mongo*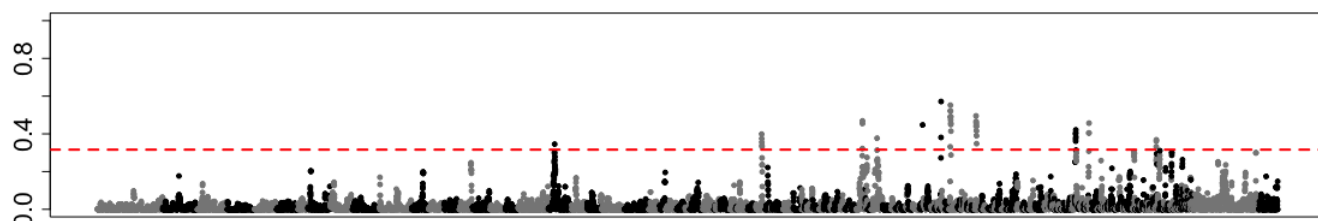*S. mariae*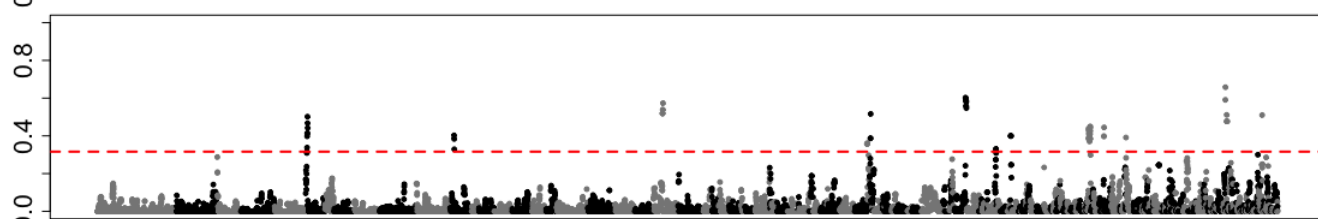*S. pindu*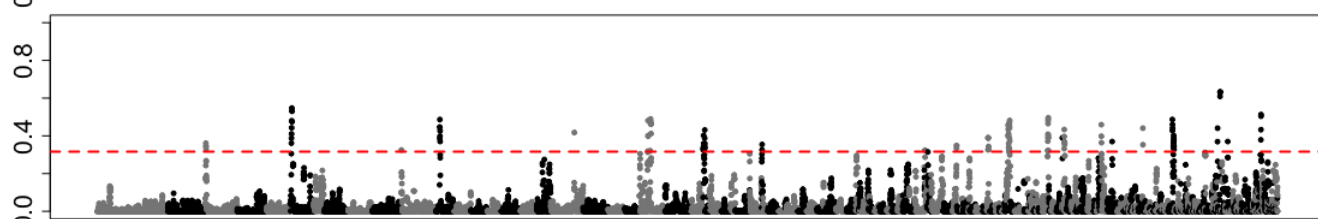

B

*K. dikume*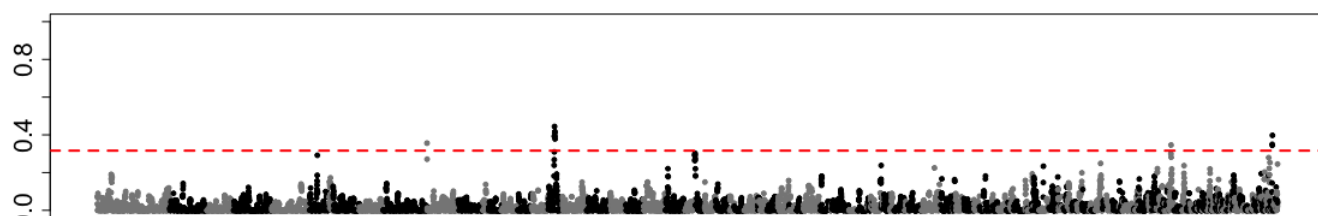*K. eisenrauti*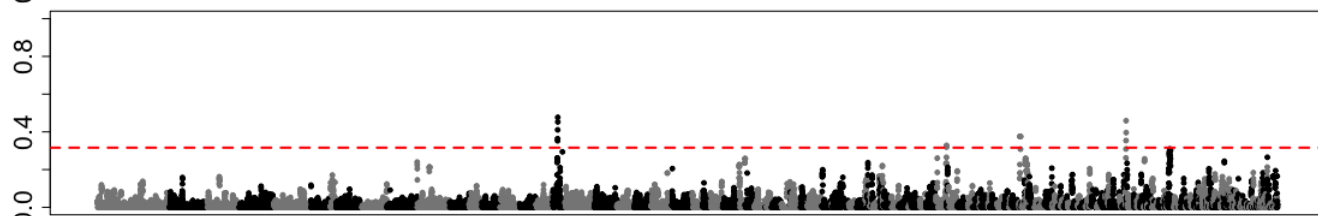*P. maclareni*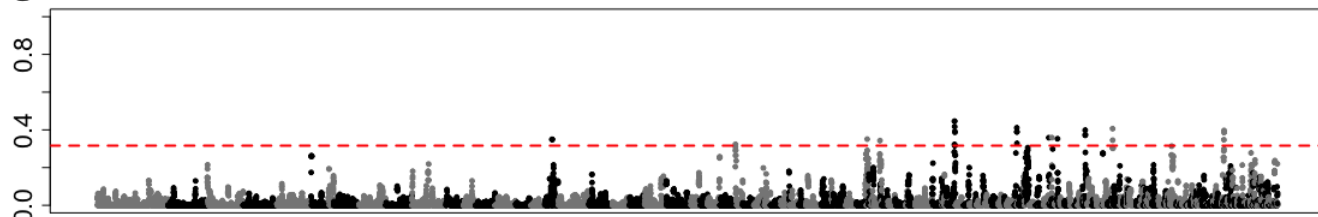

C

*M. myaka*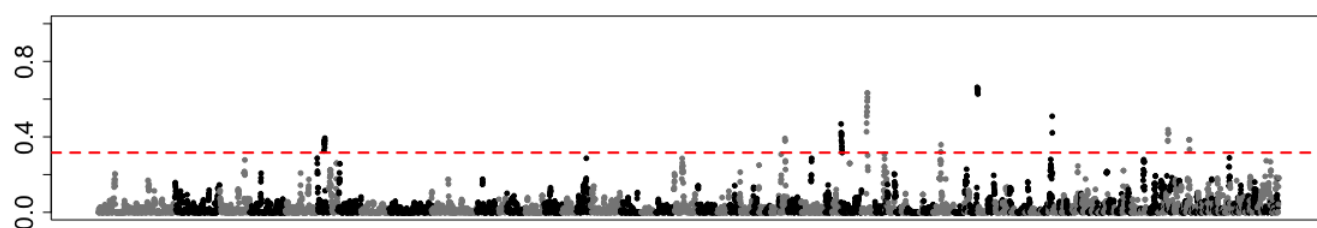

Supplement: Supplementary file 7 — Figure S6. Visualization of introgression with S. galilaeus MM across unassigned scaffolds for Barombi Mbo. [file EVL3-2-524-s007.pdf]

A

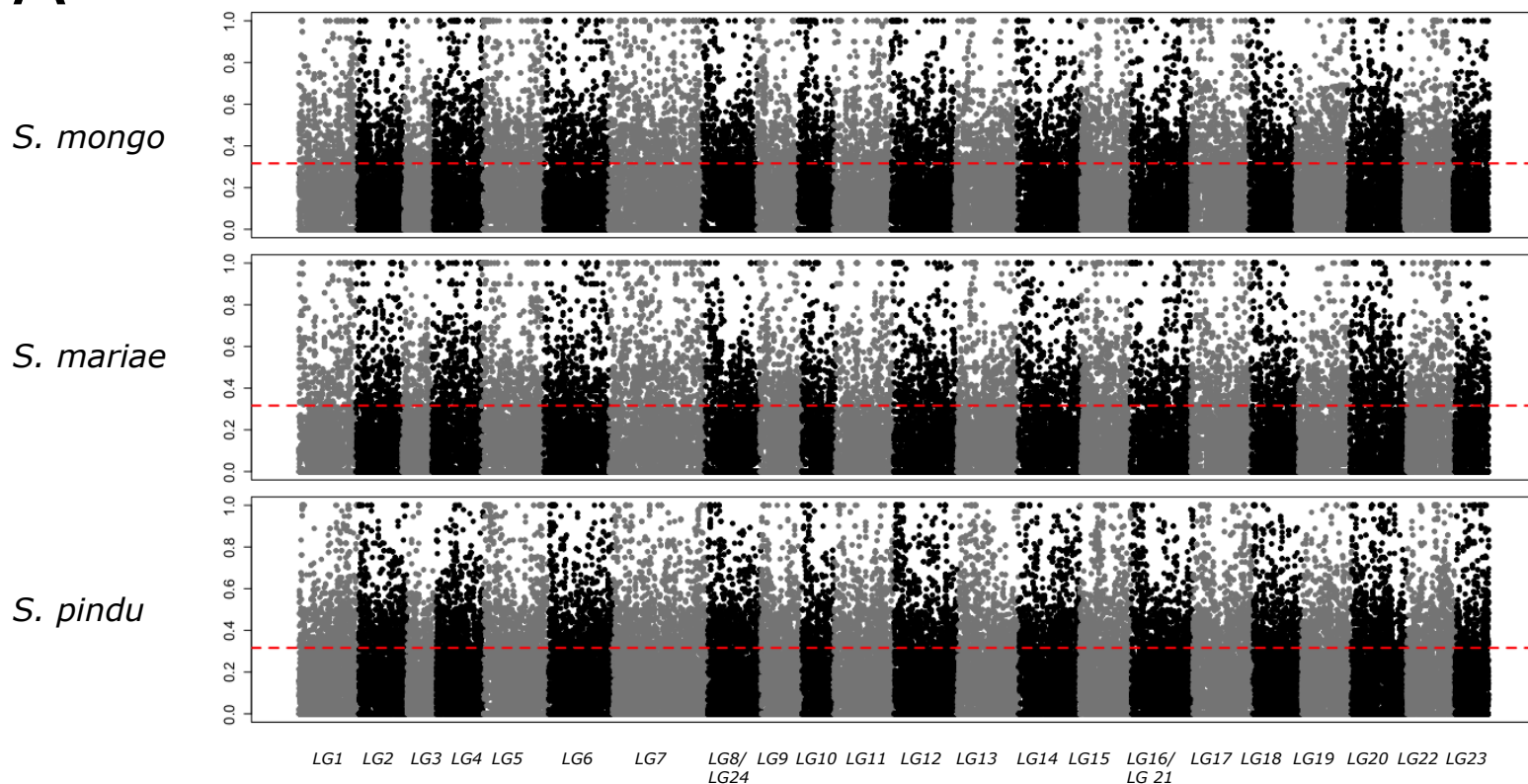

B

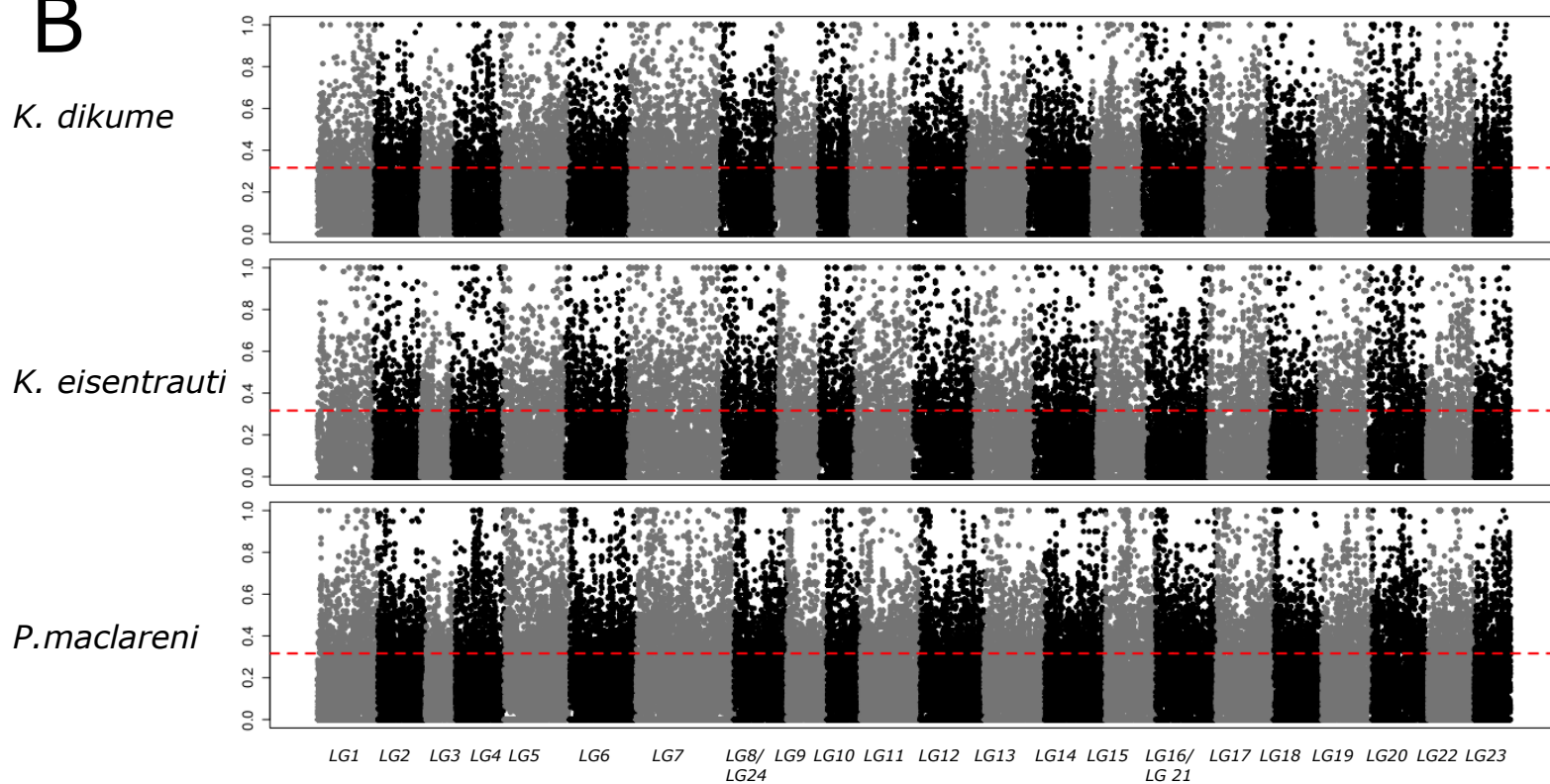

C

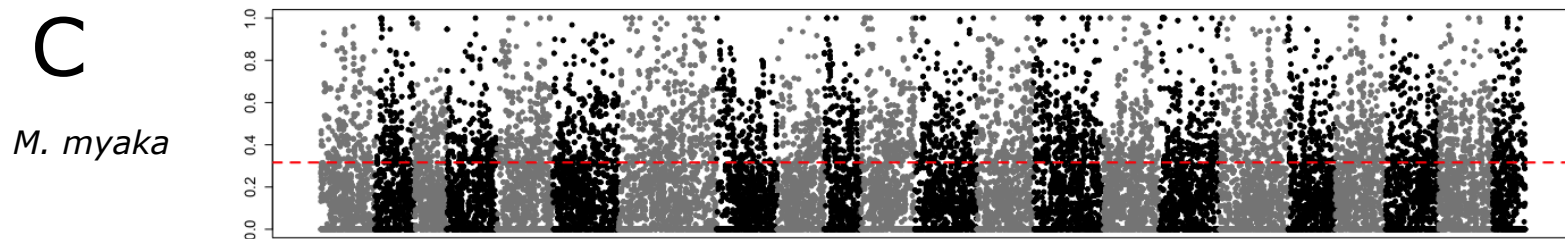

Supplement: Supplementary file 8 — Figure S7. Visualization of introgression with S. galilaeus CR across linkage groups for Barombi Mbo. [file EVL3-2-524-s008.pdf]

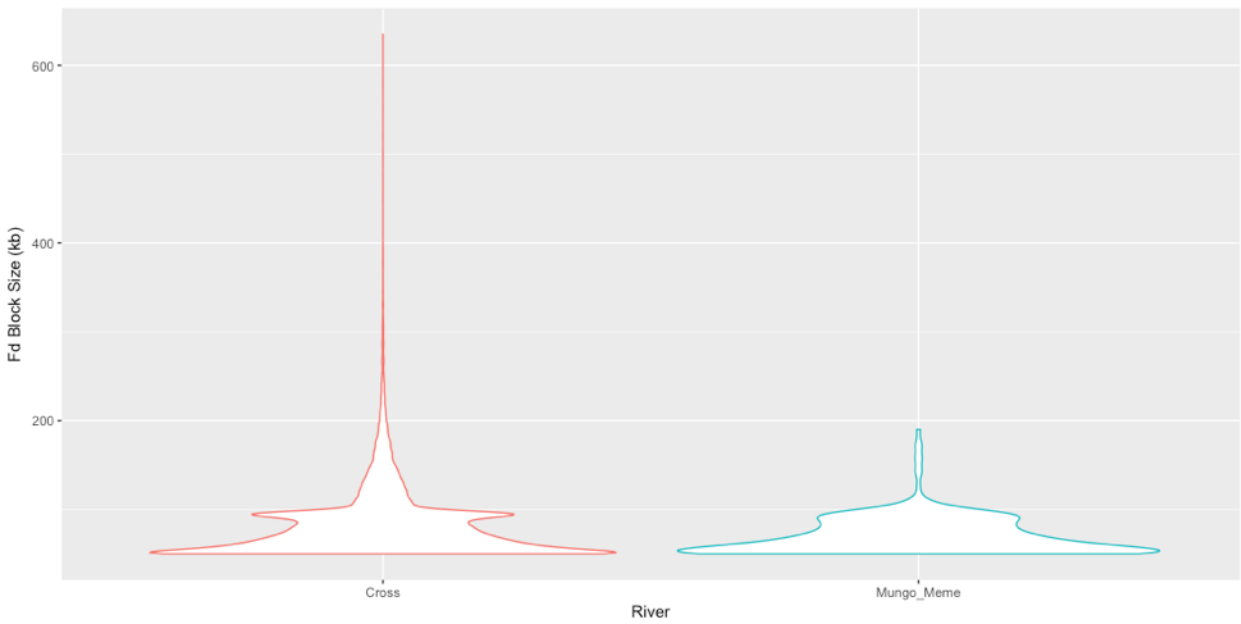

Supplement: Supplementary file 9 — Figure S8. Distribution of introgression block sizes detected from sliding window fd statistic tests for Barombi Mbo species. [file EVL3-2-524-s009.pdf]

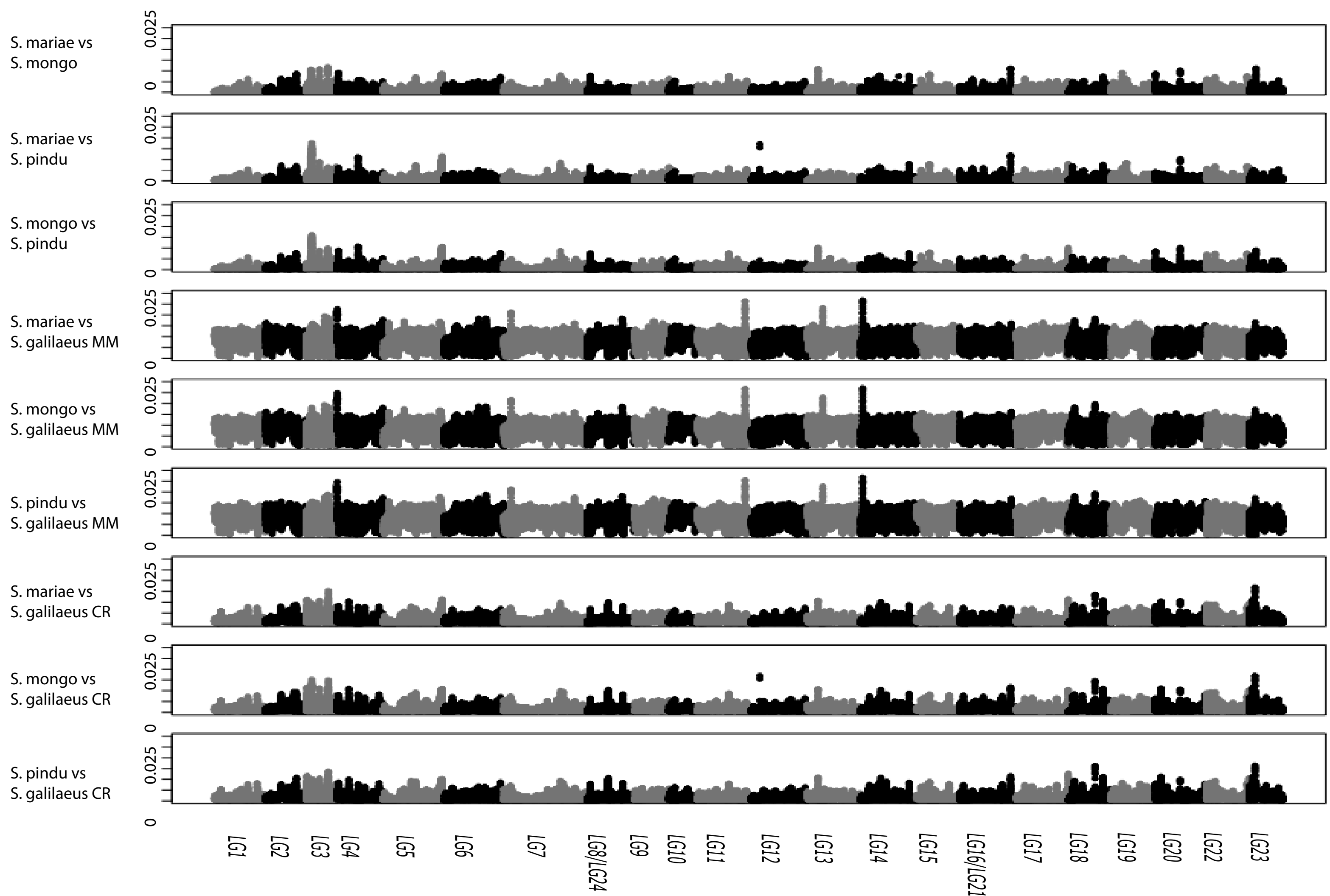

Supplement: Supplementary file 10 — Figure S9. Visualization of divergence across the genome for species of Stomatepia and S. galilaeus populations from Mungo/Meme River (MM) and Cross River (CR). [file EVL3-2-524-s010.pdf]

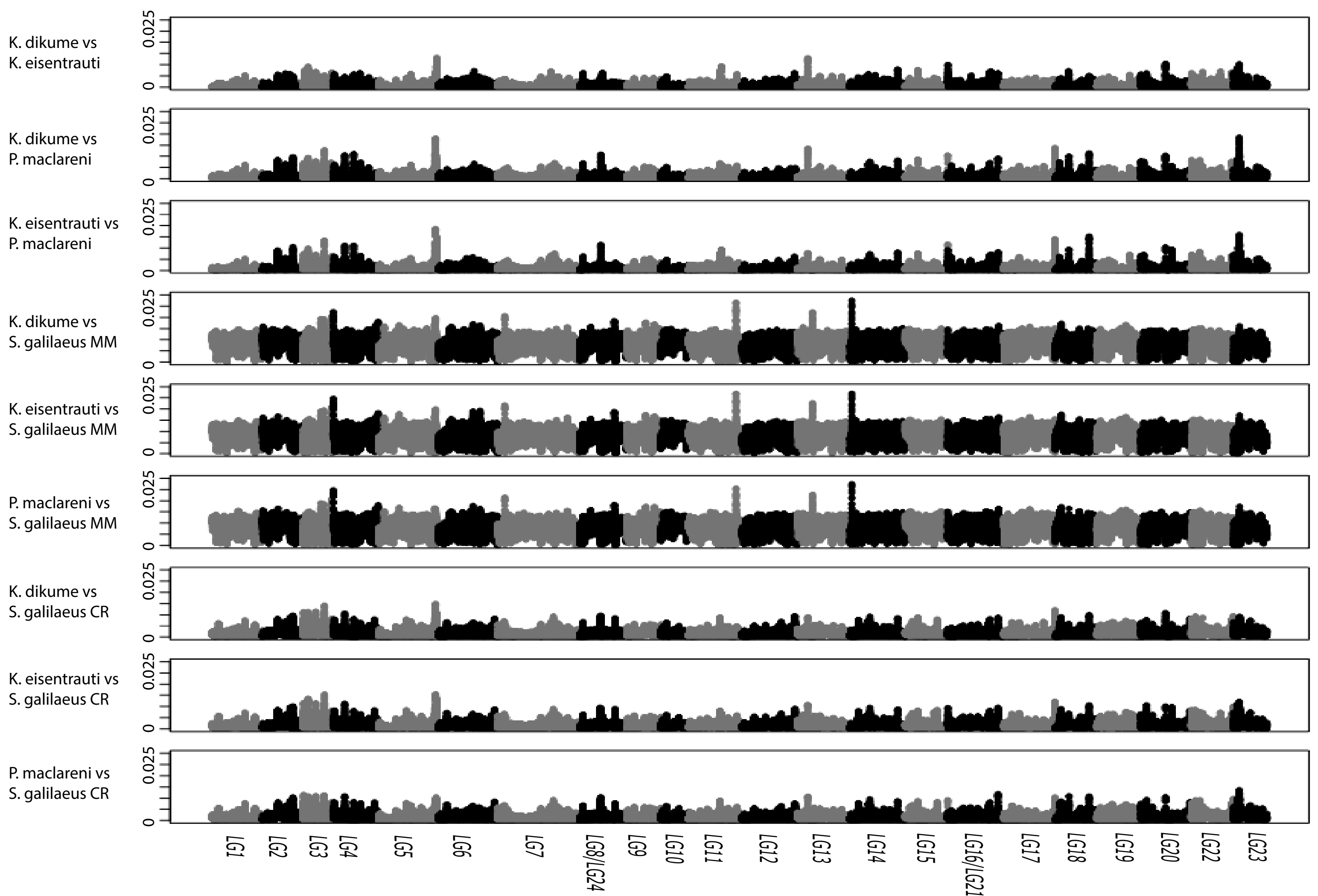

Supplement: Supplementary file 11 — Figure S10. Visualization of divergence across the genome for species of Konia and S. galilaeus populations from Mungo/Meme River and Cross River. [file EVL3-2-524-s011.pdf]

M. myaka vs  
S. linnelli

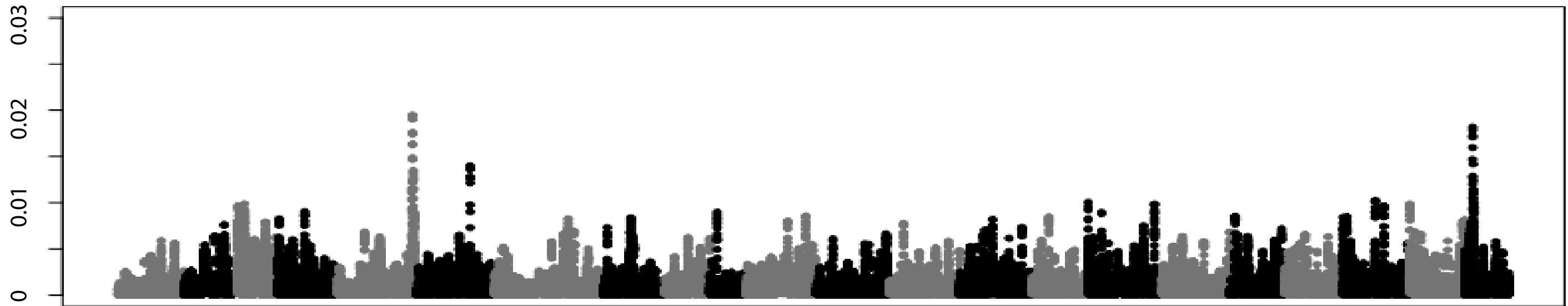

M. myaka vs  
S. galilaeus MM

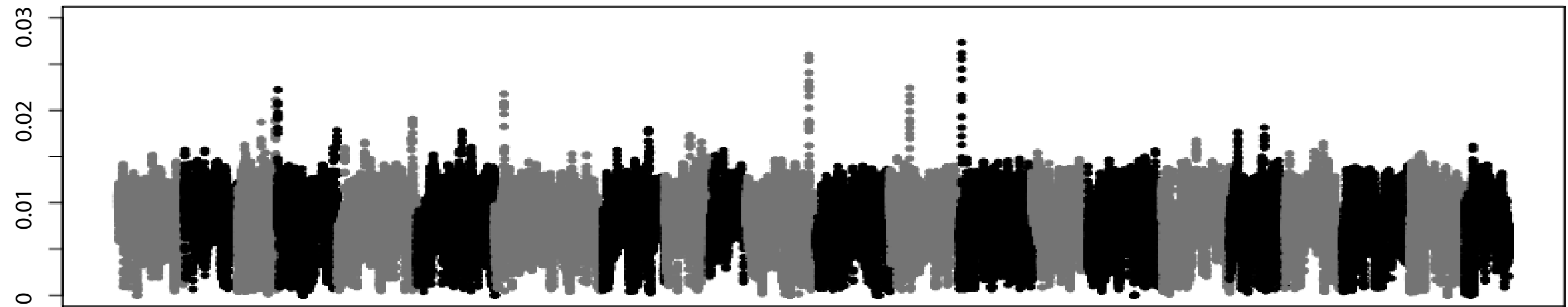

M. myaka vs  
S. galilaeus CR

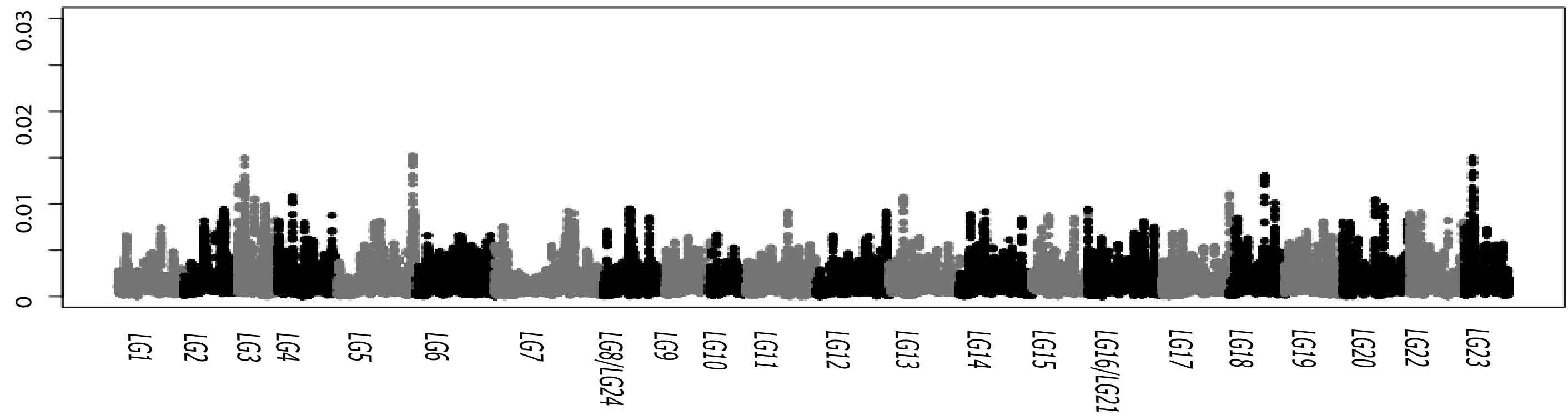

Supplement: Supplementary file 12 — Figure S11. Visualization of divergence across the genome between M. myaka and S. linnelli, S. galilaeus populations from Mungo/Meme River, and Cross River. [file EVL3-2-524-s012.pdf]

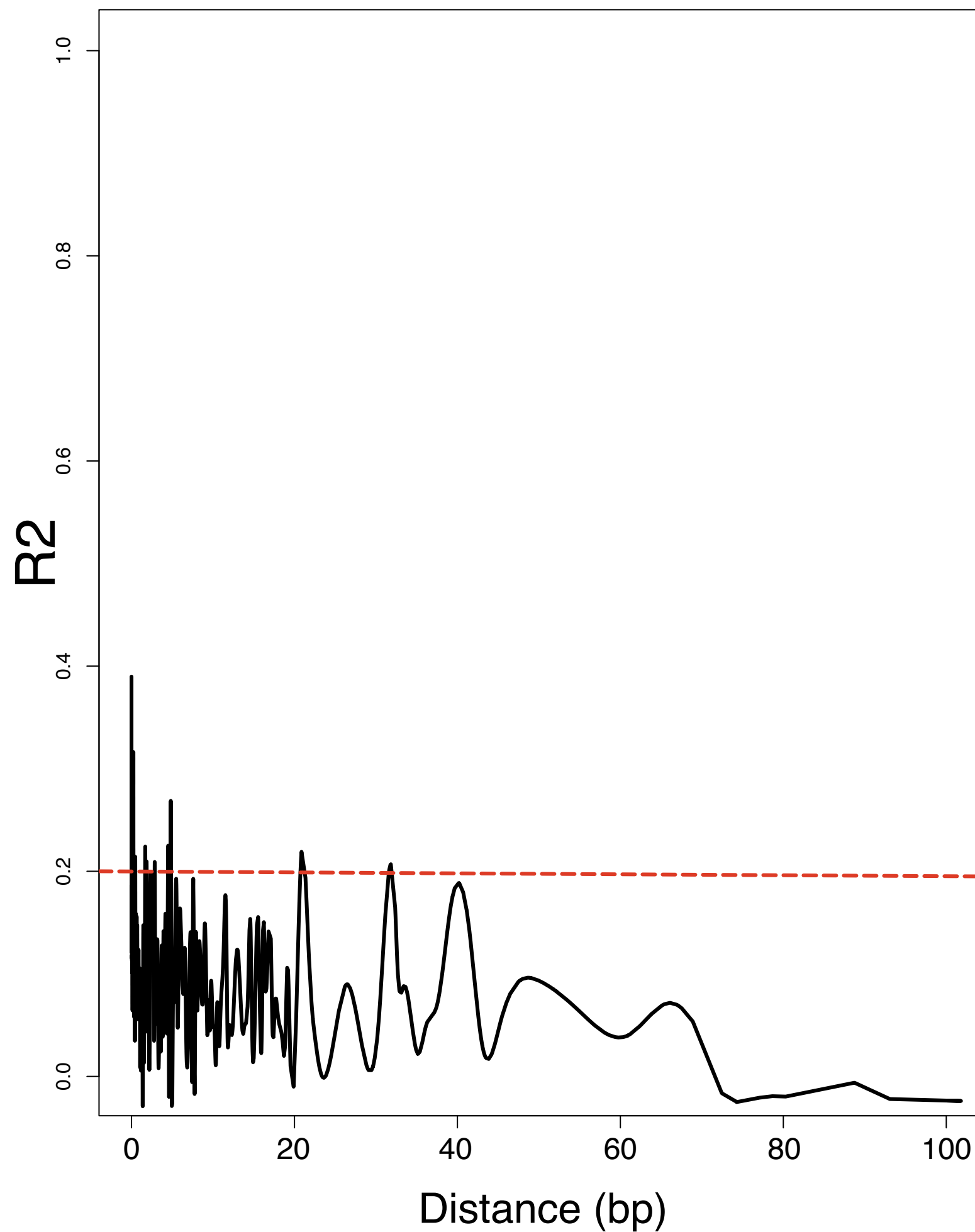

Supplement: Supplementary file 13 — Figure S12. Linkage disequilibrium decay among individuals used in this study. [file EVL3-2-524-s013.pdf]

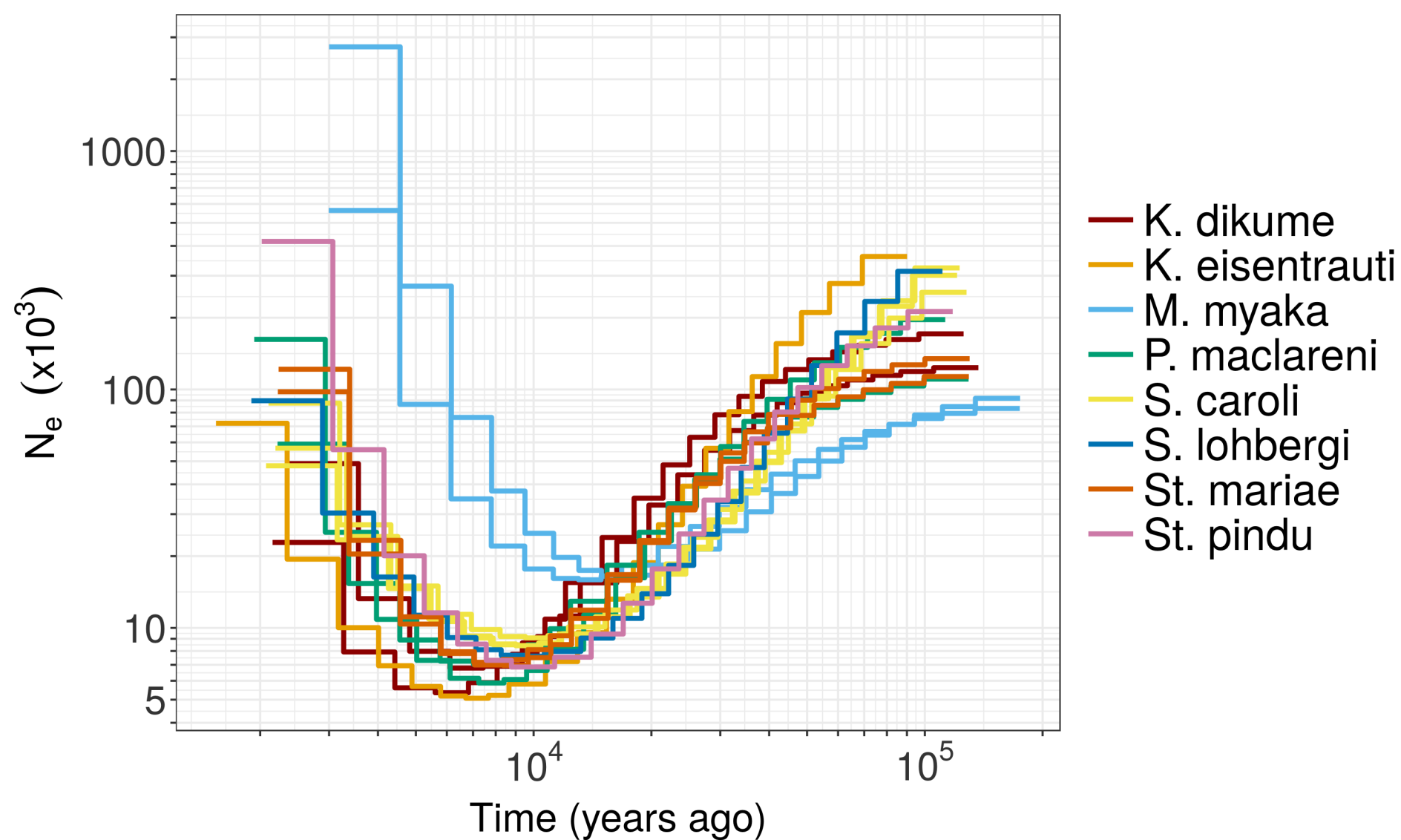

Supplement: Supplementary file 14 — Figure S13. Ancestral Population Size of Barombi Mbo Species. [file EVL3-2-524-s014.pdf]

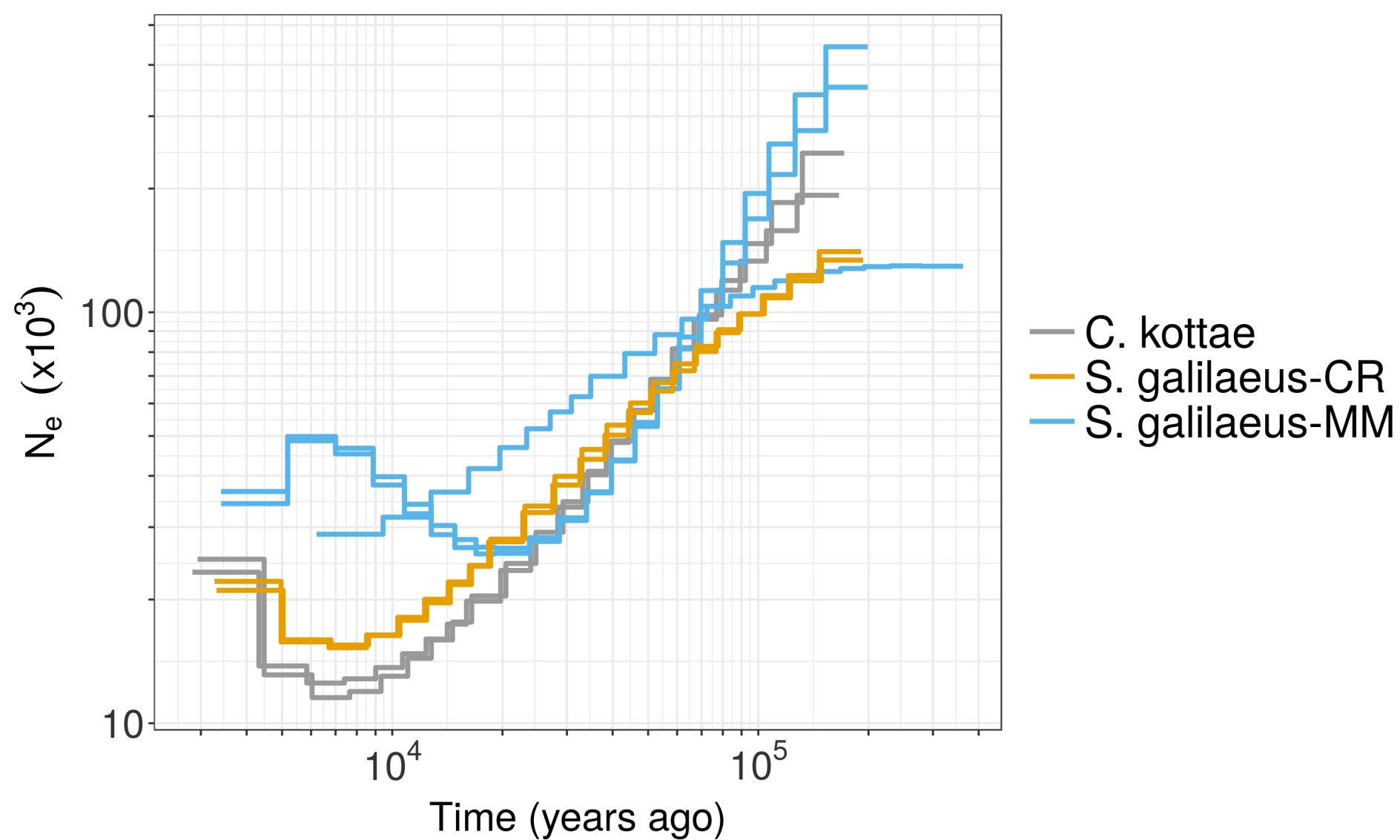

Supplement: Supplementary file 15 — Figure S14. Ancestral population size of outgroups lineages. [file EVL3-2-524-s015.pdf]
